# Evidence of DNA methylation heterogeneity and epipolymorphism in kidney cancer tissue samples

**DOI:** 10.1038/s41388-024-03270-3

**Published:** 2025-01-17

**Authors:** Sabrina H. Rossi, Victoria Dombrowe, Laura Godfrey, Teodora Bucaciuc Mracica, Sara Pita, Toby Milne-Clark, Justicia Kyeremeh, Gahee Park, Christopher G. Smith, Radoslaw P. Lach, Anne Babbage, Anne Y. Warren, Thomas J. Mitchell, Grant D. Stewart, Roland Schwarz, Charlie E. Massie

**Affiliations:** 1 Early Cancer Institute, Cancer Research UK Cambridge Centre, Cambridge Biomedical Campus, Cambridge, UK; 2 Department of Surgery, University of Cambridge, Cambridge Biomedical Campus, Cambridge, UK; 3 CRUK Cambridge Centre, University of Cambridge, Cambridge Biomedical Campus, Cambridge, UK; 4 Institute for Computational Cancer Biology (ICCB), Centre for Integrated Oncology (CIO), Cancer Research Centre Cologne Essen (CCCE), Faculty of Medicine and University Hospital Cologne, University of Cologne, Cologne, Germany; 5 BIFOLD—Berlin Institute for the Foundations of Learning and Data, Berlin, Germany; 6 Cancer Research UK Cambridge Institute, University of Cambridge, Cambridge, UK; 7 Department of Histopathology, University of Cambridge, Addenbrooke’s Hospital, Cambridge Biomedical Campus, Cambridge, UK

**Keywords:** Tumour heterogeneity, Epigenetics

## Abstract

Clear cell renal cell carcinoma (ccRCC) is characterised by significant genetic heterogeneity, which has diagnostic and prognostic implications. Very limited evidence is available regarding DNA methylation heterogeneity. We therefore generate sequence level DNA methylation data on 136 multi-region tumour and normal kidney tissue from 18 ccRCC patients, along with matched whole exome sequencing (85 samples) and gene expression (47 samples) data on a subset of samples. We perform a comprehensive systematic analysis of heterogeneity between patients, within a patient and within a sample. We demonstrate that bulk methylation data may be deconvoluted into cell-type-specific latent methylation components (LMCs), and that LMC1, which is likely to represent T cells, is associated with prognostic parameters. Differential epipolymorphism was noted between ccRCC and normal tissue in the promoter region of genes which are known to be associated with kidney cancer. This was externally validated in an independent cohort of 71 ccRCC and normal kidney tissues. Differential epipolymorphism in the gene promoter was a predictor of gene expression, after adjusting for average methylation. This represents the first evaluation of epipolymorphism in ccRCC and suggests that gains and losses in methylation disorder may have a functional relevance, gleaning important information on tumourigenesis.

## Introduction

Clear cell renal cell carcinoma (ccRCC) is the most common renal malignancy. The study of tumour heterogeneity in ccRCC has predictive and prognostic utility and has been identified as a research priority in kidney cancer [1]. ccRCC is characterised by marked genetic heterogeneity: 73% of driver mutations were not identifiable in all multi-region samples from the same patient [2, 3] and 75% of driver somatic copy number aberrations (SCNAs) demonstrated heterogeneity across multi-region samples. Both *BAP1* mutations and chromosome 9p loss are predictors of poor prognosis in ccRCC and are frequently subclonal [2]. The degree of intratumoral heterogeneity (ITH) and evolutionary pattern has been suggested to have prognostic potential in ccRCC [3, 4]. Tumour grade, the presence of necrosis and rhabdoid morphology vary across a tumour [5]. Higher architectural ITH (i.e. greater number of different patterns) was associated with higher grade, larger tumour size and more advanced stage [6].

The high degree of genetic and morphological heterogeneity has several implications. Genetic ITH may hamper the identification and validation of prognostic risk scores. ITH has been noted in 80% of patients evaluated using ClearCode34, the most commonly used prognostic risk score based on gene expression, meaning multi-region samples from the same patient were classified as high and low risk; therefore limiting use in clinical practice [7]. Analysing a small number of multi-region samples could limit our understanding of tumorigenesis by underestimating the prevalence of driver mutations or leading to an ‘illusion of clonality’, where a mutation may appear to be clonal if the analysis is limited to a small number of samples, whereas sampling a larger number of regions would reveal it to be subclonal [3]. An improved understanding of tumour evolution may offer insights into renal carcinogenesis and biomarker selection.

**Fig. 1:**
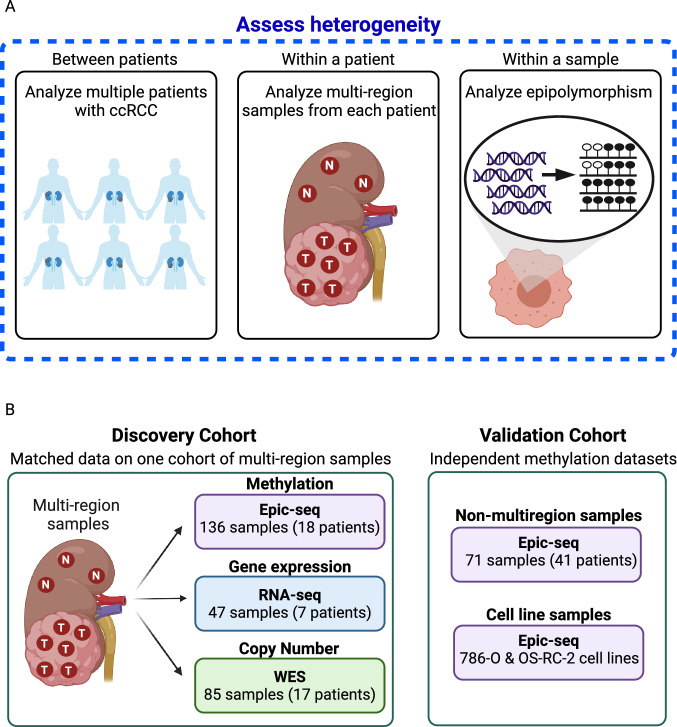
Schematic representation of samples and analysis. Heterogeneity was evaluated between patients, within a patient and within a sample (**A**). The discovery cohort consists of multi-region samples from patients with ccRCC, with matched Epic-seq, RNA-seq and WES (**B**). In addition, methylation data were generated for a cohort of tumour and normal samples from patients with ccRCC (i.e. not multi-region samples) and cell lines.

Due to the relatively low number of genetic mutations observed in ccRCC, there is a growing interest in DNA methylation as this represents an early event in tumorigenesis, with highly recurrent sets of changes [8]. Only a very small number of studies have been performed assessing methylation ITH to date, and these suggest a degree of relative methylation homogeneity within a patient, with heterogeneity between patients [9–11]. Existing studies are limited by relatively small sample sizes, absence of sequence level methylation data and absence of gene expression data, which precludes an assessment of the functional relevance of DNA methylation changes. We therefore sought to explore DNA methylation heterogeneity between patients, within a patient and within a sample aiming to improve our understanding of kidney cancer evolution and disease biology (Fig. 1A). We include matched copy number data, allowing a comparison between phylo-epigenetic and phylogenetic trees. Additionally, our work is the first evaluation of sequence level methylation data, which enables an assessment of epipolymorphism. Epipolymorphism is defined as the probability that two epialleles randomly sampled from the locus differ from each other, which represents a measure of epigenetic heterogeneity within a single sample [12]. We demonstrate that gains and losses in methylation epipolymorphism may have a functional relevance in ccRCC, gleaning important information on tumour biology. Lastly, we identify prognostic implications of methylation heterogeneity.

## Results

### Multi-region based genomic, epigenomic and transcriptional profiling of ccRCC

DNA methylation (Epic-seq; *N* = 136) data were obtained on normal kidney and multi-region tumour samples derived from 18 ccRCC patients (Fig. 1B and Supplementary Table S1). For a subset of these samples, matched whole exome sequencing (WES; 85 samples from 17 patients) and RNA-seq (47 samples from 7 patients) were generated (Supplementary Table S1). Subsequently, the results were validated on three methylation datasets: 786-O and OS-RC-2 ccRCC cell lines and an independent cohort of 71 non-multi-region samples from ccRCC patients (30 normal kidney and 41 ccRCC samples; Supplementary Table S2 and Fig. 1B).

### Greater methylation heterogeneity is observed between patients than within patients

Assessing all available CpGs (N = ~1.1 million CpGs) samples cluster by pathological subtype, with more variability in tumour compared to normal kidney (Supplementary Figure S1A). Overall, in the majority of cases there is more inter- than intra- tumoural heterogeneity, and this is consistent despite using different cut-offs (evaluating the top 10,000 and top 50,000 CpGs with the highest variance in tumour samples), in keeping with limited available evidence [9–11, 13] (Supplementary Figure S1B–D). Patient 7068 was the notable exception, and this individual appears to have the highest ITH (Supplementary Figure S1B). Epigenetic age was significantly higher in ccRCC compared to normal tissue (median methylation age 63 vs 59 years, *p* value < 0.01, Wilcoxon test) and this finding was also confirmed in TCGA samples (Supplementary Figure S2). Accelerated epigenetic ageing has been associated with increased cancer risk, as well as prognosis in ccRCC [9], chromophobe RCC [14] and other cancers. In the majority of patients, the predicted to chronological age ratio (PCAR) was similar amongst tumour samples derived from the same patient (maximum difference in PCAR amongst tumour samples <30%) (Supplementary Figure S2), suggesting relative methylation homogeneity within a patient.

### Variable resemblance between genetic, epigenetic and transcriptomic ITH

Mutation analysis revealed *VHL* and *PBRM1* as the most commonly mutated genes, identifying other frequently mutated genes (including *SETD2* and *KDM5C*), in keeping with the existing literature (Supplementary Figure S3A) [15]. As expected, chromosome 3p was the most common CNA (loss in 76% of samples) [16]. *VHL* was clonally mutated in all but two patients; patient 5826 and 7281 (mutation frequency 93%). Patient 5826 demonstrated hypermethylation in selected CpGs located in the *VHL* promoter in the absence of a *VHL* mutation, suggesting methylation may be a putative mechanism of *VHL* inactivation in this case (Supplementary Figure S3B). However, this was not noted for patient 7281. Differential methylation was noted in tumour versus normal samples for CpGs located near *PBRM1* and *SETD2* genes, however this did not bear any association with the genes’ mutational status. Unlike El Khoury et al. [13], there was no significant global methylation change in *SETD2* mutant versus wild type tumours (*p* > 0.05, Wilcoxon test).

We calculated the Average Pairwise ITH (APITH) index, a validated metric to quantify intra-tumoral heterogeneity independently of the number of tumour samples evaluated [17]. We compared the APITH index derived using methylation and SCNA data respectively, to directly compare genetic and epigenetic ITH. There was no correlation between the methylation APITH and the copy number APITH (Pearson correlation coefficient = 0.33, *p* value = 0.216, correlation test; Supplementary Figure S4). There was no significant association between methylation APITH nor SCNA APITH and PCAR nor any clinical/prognostic parameters. Interestingly, methylation APITH scores were previously shown to be associated with overall survival and risk of distant metastases in lung cancer [17], but no association was noted in papillary RCC [18].

**Fig. 2:**
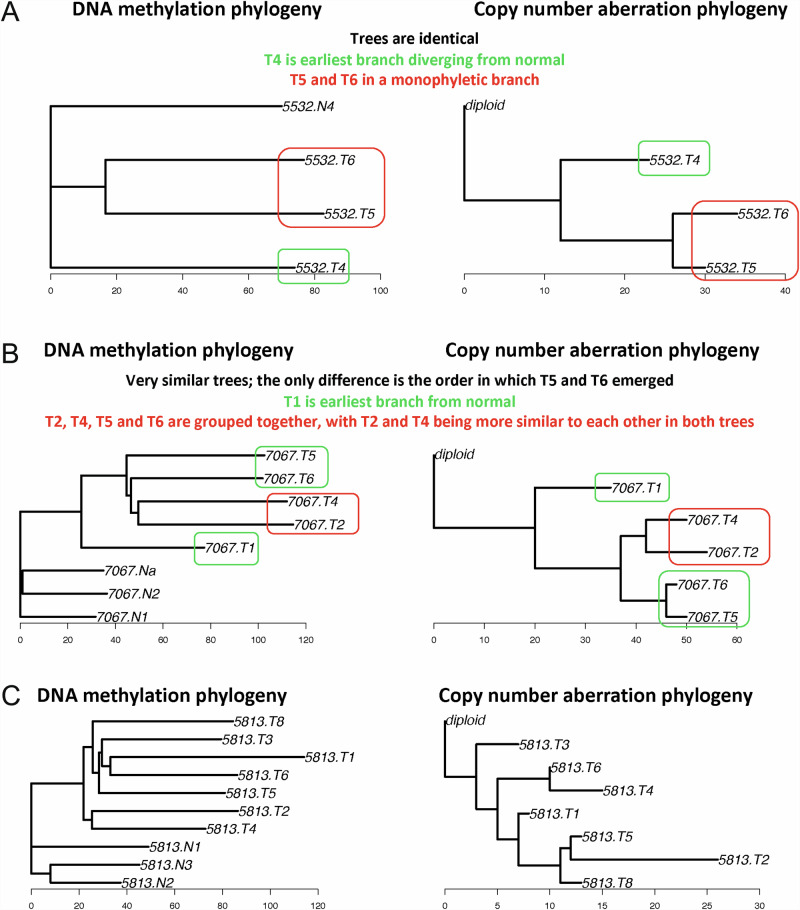
Genetic and epi-genetic phylogenies for patients. Phylogenies using DNA methylation and copy number data are compared for each patient, for patients 5532 (**A**), 7067 (**B**) and 5813 (**C**). Multi-region tumour samples are denoted with a *T*, where each sample is labelled numerically to demarcate tumours from the same patient. Normal samples as denoted with an *N*. Monophyletic clades which are present in both phylo-epigenetic and phylogenetic trees are shown in red, with other similarities shown in green. In patients 5532 and 7067 extremely similar methylation and SCNA trees were noted, suggesting potential co-evolution, whereas 5813 demonstrated distinctly different phylogenetic and phylo-epigenetic trees, indicating that potentially methylation and SCNA changes may have evolved separately.

For each patient with >4 multi-region tumour samples (*N* = 8), phylogenies were inferred using methylation data and SCNA data respectively, and the phylo-epigenetic and phylogenetic trees were compared using the Robinson-Fould measure (Supplementary Table S3). In two patients (i.e. 5532 and 7067) extremely similar methylation and SCNA trees were noted, suggesting potential co-evolution (Fig. 2). Patients 5842, 6300 and 6262 had some consistent similarities between epigenetic and genetic trees, although some differences were also noted (Supplementary Figures S5 and S6). Conversely, other patients (for example 5644, 5813 and 6285) demonstrated distinctly different phylogenetic and phylo-epigenetic trees, indicating that potentially methylation and SCNA changes may have evolved separately (Fig. 2 and Supplementary Figures S5 and S6).

**Fig. 3:**
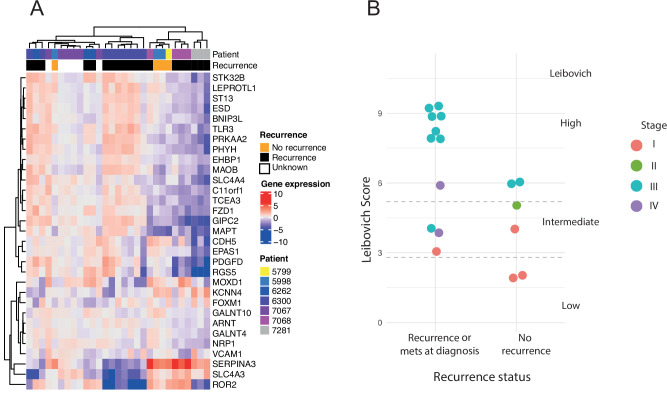
Prognostic scores in ccRCC. Gene expression is visualised for genes which contribute to the Clearcode34 prognostic risk score (**A**). Expression was derived from RNA-seq and is median centred and presented on a log2 scale. Multi-region samples from the same patient are shown, along with recurrence status. Leibovich score for patients that did not have a recurrence versus patients who either had metastases at diagnosis or developed recurrence on follow up (**B**). Each dot represents a patient, where the colour of the dot shows tumour stage (stage I-IV). Individual Leibovich scores can be aggregated into three groups: low (0–2), intermediate (3–5) and high (≥6).

We evaluated transcriptional heterogeneity by assessing the validated ClearCode34 [19, 20] prognostic risk score in multi-region ccRCC tissue samples using RNA-seq (Fig. 3A). Although samples from the same individual tend to cluster together in most cases, there are several multi-region samples which do not cluster by patient (for example samples from 5998, 7067 and 7068). This is in keeping with available evidence suggesting that multi-region samples from the same patient can have different expression patterns for ClearCode34 genes [7], and demonstrates evidence of transcriptional heterogeneity. Expression of ClearCode34 genes was not able to accurately distinguish between patients with and without a recurrence, though the analysis was limited by the small sample size. We also assessed the CpG island methylator phenotype (CIMP), an established prognostic marker based on methylation data [21]. The CIMP was able to differentiate tumour from normal samples, but was unable to differentiate patients who had a recurrence from those that did not (Supplementary Figure S7).

Given no obvious associations were found between methylation, transcriptional ITH or prognostic parameters, we sought to evaluate whether the patients’ recurrence status could be recapitulated using the Leibovich score, which is used clinically (Fig. 3B). Whilst the Leibovich score performed well in low and high-risk categories, it performed less well in intermediate risk. In this cohort, 0% of patients (*N* = 0/2) with a low Leibovich score (score 0–2) developed a recurrence, whilst 100% of patients with a Leibovich score ≥8 had a recurrence. 60% (*N* = 3/5) of individuals with an intermediate score (score 3–5), developed a recurrence, highlighting the need for better risk stratification. It is therefore not surprising that there was no obvious association between clinical parameters and methylation and SCNA ITH in this smaller dataset.

### Significant epipolymorphism is noted in genes known to be associated with renal cell carcinoma

**Fig. 4:**
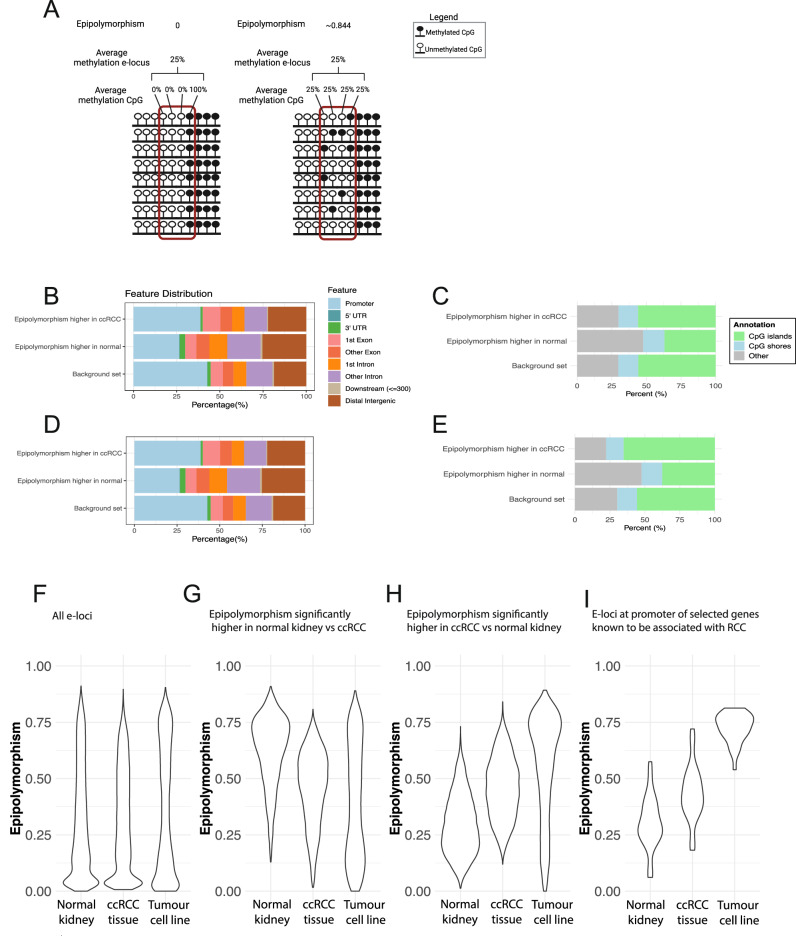
Epipolymorphism in normal kidney, ccRCC tissue and 786-O ccRCC cell line. Schematic explanation of epipolymorphism and average methylation (**A**). Lollipops represent individual CpGs (black: methylated, white: unmethylated) and an e-locus is defined as four adjacent CpGs (red rectangle). The diagram demonstrates average methylation levels at each CpG, average methylation levels across an e-locus and epipolymorphism. Epipolymorphism measures how variable the methylation pattern is within and between reads. This explains how two samples may have the same average methylation across an e-locus, but different epipolymorphism values. This figure was adapted from [12]. Annotation for e-loci with significant differential epipolymorphism in ccRCC versus normal kidney tissue in the discovery cohort (135 samples; **B**, **C**). Annotation for e-loci with significant differential epipolymorphism in ccRCC versus normal kidney in the validation cohort (71 samples; **D**, **E**). Epipolymorphism is shown in ccRCC tumour tissue, normal kidney tissue and the 786-O renal cancer cell line for: all e-loci (**F**), e-loci with significantly higher epipolymorphism in normal kidney (**G**) and e-loci with significantly higher epipolymorphism in ccRCC (**H**). **I** demonstrates epipolymorphism values for selected e-loci in the promoter region of 8 genes which are known to be associated with kidney cancer and were found to have significantly higher epipolymorphism in ccRCC vs normal kidney. These genes were selected to be visualised as an illustrative example.

We evaluated epipolymorphism and assessed the possible impact on gene expression. For each locus of 4 adjacent CpGs (thereby referred to as an epigenetic locus; e-locus) we derived epipolymorphism and average methylation for ccRCC tumour and normal samples, as previously described [12] (Fig. 4A)(*N* = 138,412 e-loci, for 135 multi-region samples). The majority of e-loci have fully concordant methylation, in keeping with the existing literature [12] (Supplementary Figure S8). 28,300 e-loci demonstrated significant differential epipolymorphism in ccRCC versus normal tissue, with 14,418 e-loci being significantly higher in ccRCC, and 13882 e-loci having significantly higher epipolymorphism in normal tissue (absolute epipolymorphism difference >0.1 and adjusted *p* value < 0.01). E-loci with significantly higher epipolymorphism in ccRCC were located more commonly at gene promoters and in CpG islands, than e-loci with significantly higher epipolymorphism in normal kidney (34% vs 27% at gene promoters; *p* value < 2.2e-16, and 56% vs 37% in CpG islands; *p* value < 2.2e-16; proportions test) (Fig. 4B, C). These findings were externally validated in an independent cohort of 71 normal kidney and ccRCC samples. E-loci that had significantly higher epipolymorphism in ccRCC were located more commonly at gene promoters and in CpG islands, than e-loci with significantly higher epipolymorphism in normal kidney (39% vs 26% at gene promoters; *p* value < 2.2e-16, and 65% vs 38% in CpG islands; *p* value < 2.2e-16; proportions test) (Fig. 4D, E).

Gene set enrichment analysis (GSEA) was performed for e-loci with significant differential epipolymorphism located in gene promoter regions. A significant enrichment was observed for the following pathways: *Wnt* signalling (e.g. *MYC*, *WIF1* and *UBC*), cell junction organisation (including claudins, keratins, *CDH* genes, *SDK1*), solute-carrier membrane (*SLC*) and voltage-gated potassium channel (*KCN*) genes (all adjusted *p* values < 0.05). Disease ontology analysis revealed a significant enrichment for genes known to be associated with renal cell carcinoma (47 out of 557 genes *vs* 380 out of 8007 genes in the background set, chi squared adjusted *p* value = 0.025) (Supplementary Table S4). These findings were externally validated in an independent cohort of ccRCC tissue samples, and subsequently in kidney cancer cell line data. 31 genes demonstrated differential epipolymorphism in the external validation cohort (at the same e-loci as the discovery cohort) (Supplementary Table S4). This suggests that although disordered methylation may be a stochastic process which occurs throughout the genome, ccRCC is associated with gains and losses of locally disordered methylation at the promoter region of known ccRCC genes.

Evaluating e-loci with differential epipolymorphism in ccRCC vs normal kidney revealed that, as expected, the distribution of epipolymorphism values in the 786-O cancer cell line was more similar to ccRCC (and generally higher than ccRCC) compared to normal tissue (Fig. 4F–H). This suggests that disordered methylation within a pure cell line may be associated with tumorigenesis, rather than being an artefact of cell contamination. Next, epipolymorphism was visualised at e-loci within the promoter region of eight selected genes known to be associated with RCC identified from our previous analysis (*MAD2L2*, *TRPC4*, *NDRG2*, *NOS2*, *TTYH2*, *RAB37*, *FRZB* and *TERT*). Epipolymorphism values in ccRCC tissue and the cancer cell line were higher than normal kidney (Fig. 4I). The analysis was repeated for a second cell line (OS-RC-2) and similar patterns were notes, confirming our findings (Supplementary Figure S9).

### Differential epipolymorphism in the gene promoter is a predictor of gene expression

Matched Epic-seq and RNA-seq data were obtained to explore the association between gene expression, methylation and epipolymorphism (*N* = 47 multi-region samples). The analysis focused on e-loci which have significant differential epipolymorphism in ccRCC vs normal kidney and are located within the promoter region of genes, as these are more likely to be functional (*N* = 7536 e-loci).

A linear model was developed to predict gene expression based on epipolymorphism. 1870 e-loci (in the promoter region of 475 unique genes) were identified where epipolymorphism was a significant predictor of gene expression in univariate analysis (BH adjusted *p* value < 0.05). This included genes known to be associated with kidney cancer (*ALOX5*, *WT1*, *CD44*, *KRT7*, *KRT18*, *CD276*, *CXCL16*, *RAB37*, *BCL2L11*, *JAG1*, *EGF*, *HLA-A*, *IGF2BP3*, *SLC16A3* and *DPP6*). A negative correlation was seen in 87% (*N* = 976/1119) of e-loci with higher epipolymorphism in ccRCC, and 60% (*N* = 449/751) of e-loci with higher epipolymorphism in normal kidney. The correlation between epipolymorphism and gene expression was negative in the majority of cases, suggesting disordered methylation within gene promoters tends to be associated with transcriptional repression.

**Fig. 5:**
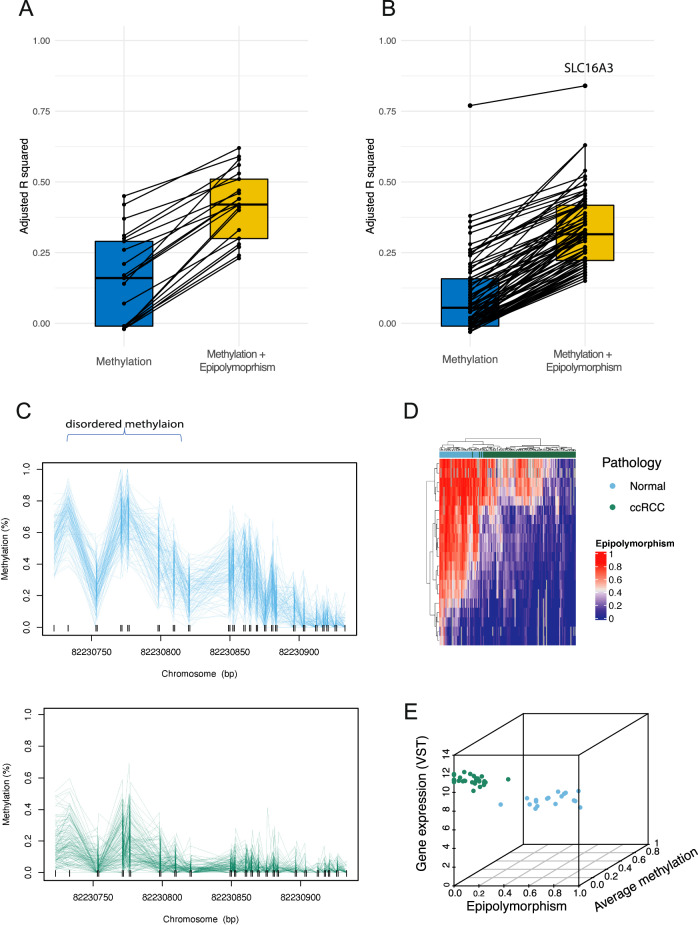
Epipolymorphism, methylation and gene expression. Adjusted *R*^2^ for a linear model to predict gene expression based on methylation alone versus methylation and epipolymorphism for genes which had a statistically significant improvement in the *R*^2^. Results are shown separately for e-loci with significantly higher epipolymorphism in ccRCC (**A**) and e-loci with significantly higher epipolymorphism in normal kidney (**B**). *SLC16A3* is the gene with the highest adjusted *R*^2^ and is an outlier, therefore it was explored more in detail in (**C**–**E**). Methylation levels along the promoter region of the *SLC16A3* gene (**C**). Heatmap demonstrating epipolymorphism in each e-locus in the promoter region of *SLC16A3*, in ccRCC and normal kidney tissue (**D**). Scatterplot of gene expression versus epipolymorphism and average methylation in a 3D scale (**E**). Epipolymorphism and average methylation are higher for normal kidney than ccRCC, and there is associated reduced expression in normal kidney. In ccRCC, there is global hypomethylation, whereas in normal kidney there is evidence of disordered methylation (hypermethylation and hypomethylation of adjacent CpGs).

To ascertain the effect of epipolymorphism beyond methylation, we subsequently evaluated a linear model predicting gene expression based on methylation alone or methylation and epipolymorphism, as previously described [22]. We thus identified 216 e-loci (at the promoter region of 103 unique genes) where the addition of epipolymorphism resulted in significant improvements in the model compared to a model based on methylation alone (i.e. significant increases in adjusted *R*^2^, likelihood ratio test BH adjusted *p* value < 0.05; Fig. 5A, B). These e-loci consist of 43 e-loci (in the promoter of 21 genes) with significantly higher epipolymorphism in ccRCC, and 173 e-loci (in the promoter of 82 genes) with significantly higher epipolymorphism in normal tissue (Supplementary Table S5 and S6). Many of the genes identified have previously been implicated in cancer disease biology and ccRCC in particular, including membrane transporters, genes involved in cell proliferation, adhesion, angiogenesis and hypoxia signalling.

*SLC16A3* was the top-ranking gene with the highest *R*^2^ value for the linear model containing methylation and epipolymorphism to predict gene expression (adjusted *R*^2^ = 0.84; Supplementary Tables S5 and S6; Fig. 5B). *SCL16A3* plays a key role in mediating the Warburg effect and tumourigenesis in ccRCC and has been linked to prognosis [23–25]. Our data reveals *SCL16A3* promoter hypomethylation in ccRCC (Fig. 5C) and an association with increased gene expression compared to normal kidney, in agreement with the available literature. We add to existing evidence by demonstrating that epipolymorphism is significantly different in 20 e-loci in the gene promoter region in ccRCC versus normal, with disordered methylation in normal tissue and ordered methylation (i.e. concordant hypomethylation) in ccRCC and this is associated with changes in gene expression (Fig. 5D, E). This could suggest that disordered methylation in the normal tissue is an early event which predisposes to hypomethylation in ccRCC and this has a functional relevance on gene expression.

### Analysis of methylation ITH enables the selection of better predictive tumour markers

Differentially methylated cytosines (DMCs) were called between ccRCC and normal in the 136 multi-region samples, with patient as a covariate (*n* = 107947 DMCs). The top 10% of DMCs with the lowest variance in tumour samples clearly separate tumour versus normal and demonstrate both low between and within sample heterogeneity, therefore representing ideal biomarker targets for diagnostic clinical applications (Supplementary Figure S10). GSEA of DMCs in the gene promoter region identified pathways associated with cell-cell communication and cell adhesion (e.g. *SDK1*), claudins (*CLDN8*, *CLDN10* and *CLDN14*) and signal regulatory protein family interactions (e.g. protein kinases such as *PTK2* and *SRC*), which are key tumorigenic pathways. This implies these DMCs are likely to represent ccRCC-specific changes.

Evaluating DMCs with the highest tumour variance, purity is significantly higher in samples that cluster more closely with normal tissue than those that cluster away from normal (mean purity 59.4% vs 39.9%, *p* value = 3.9e-8, T-test; Supplementary Figure S10). GSEA of these DMCs highlighted pathways associated with neutrophil degranulation (51/464 vs 480/10,654; adjusted *p* value = 2.28e-06). Taken together, these results suggest that a proportion of DMCs that are called as significantly different between ccRCC and normal tissue, may represent immune cell infiltration, rather than tumour-specific markers. We then proceeded to reference-free cell type deconvolution of bulk methylation data to explore these components.

**Fig. 6:**
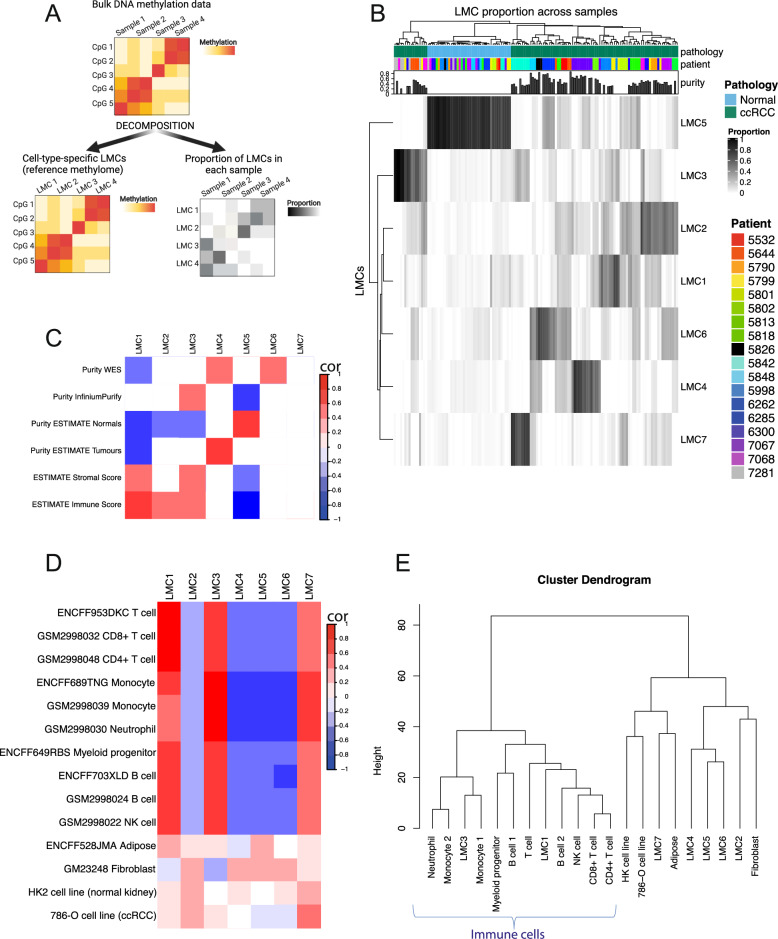
Decomposition of bulk DNA methylation data into latent methylation components. Schematic explaining how bulk DNA methylation data are decomposed into cell-type-specific latent methylation components (LMCs) and the proportion of LMCs in each sample (**A**). Heatmap demonstrating the 7 LMCs, and the proportion of each of these LMCs in the tissue samples (**B**). For each sample, the top annotation bar shows the pathology (ccRCC vs normal kidney), sample purity and patient ID (from which the sample was derived). Heatmap demonstrating correlation values (cor) for the proportion of each LMC and purity values, for tissue samples (**C**). Purity values are derived using three independent methods: WES, RNA-seq (‘ESIMATE’) and DNA methylation (‘InfiniumPurify’). ‘ESTIMATE’ is the only method which provides purity estimates for normal samples, therefore these are shown separately in the heatmap. Heatmap demonstrating correlation values (cor) between each LMC and reference methylomes for various cell types (**D**). For each cell type, the accompanying code denotes the reference from which it was derived (from publicly available reference methylomes). In (**C**, **D**) a significant positive and negative correlation are shown in red and blue respectively. White denotes no significant correlation (*p* value > 0.05). Cluster dendrogram obtained using methylomes for the seven LMCs and reference methylomes (**E**). LMC1 and LMC3 cluster with immune cells, on the left branch of the dendrogram.

Regularized non-negative matrix factorization was used to decompose bulk methylation data into two matrices: cell-type-specific latent methylation components (LMCs) and the proportion of LMCs in each sample, using ‘MeDeCom’ (Fig. 6A) [26]. Methylation data were deconvoluted into 7 LMCs, which represent different cell types and an orthogonal analysis was performed to determine each cell type (Fig. 6B). In summary, LMC5 appears to represent normal kidney epithelium, LMC4 is likely to be a marker of ccRCC, whereas LMC1 and LMC3 are likely to represent immune cells (LMC2 and LMC6 remain unclear; Supplementary Table S7). LMC5 was positively correlated with RNA-seq purity estimates in normal tissue, but not ccRCC tumours, suggesting LMC5 represents normal kidney (Fig. 6C). In tumours, LMC5 was negatively correlated with purity estimates from ‘InfiniumPurify’, which determines tumour purity as a function of contamination with normal tissue (Fig. 6C; Supplementary Figure S11). LMC4 was correlated with RNA-seq purity estimate for tumours, but not for normal kidney, and was also positively correlated with purity estimates derived by WES (Fig. 6C). LMC4 therefore appears to represent kidney epithelium in ccRCC tumours. LMC1 was highly positively correlated with the reference methylome for T cells derived from two separate sources (correlation = 0.83, adjusted *p* value < 0.001, correlation test), suggesting it may indeed represent this cell type (Fig. 6D, E). This was corroborated by the fact that the proportion of LMC1 in each tissue sample correlated with the immune score calculated using RNA-seq using ESTIMATE [27] (Supplementary Table S7). LMC3 was also positively correlated with the immune score and had a strong correlation with the reference methylome for monocytes and neutrophils, meaning it may indeed represent tumour-associated macrophages (Fig. 6D, E). Tumour samples from the same patient tend to cluster together, suggesting higher heterogeneity between than within patients, as already noted in Supplementary Figure S1.

**Fig. 7:**
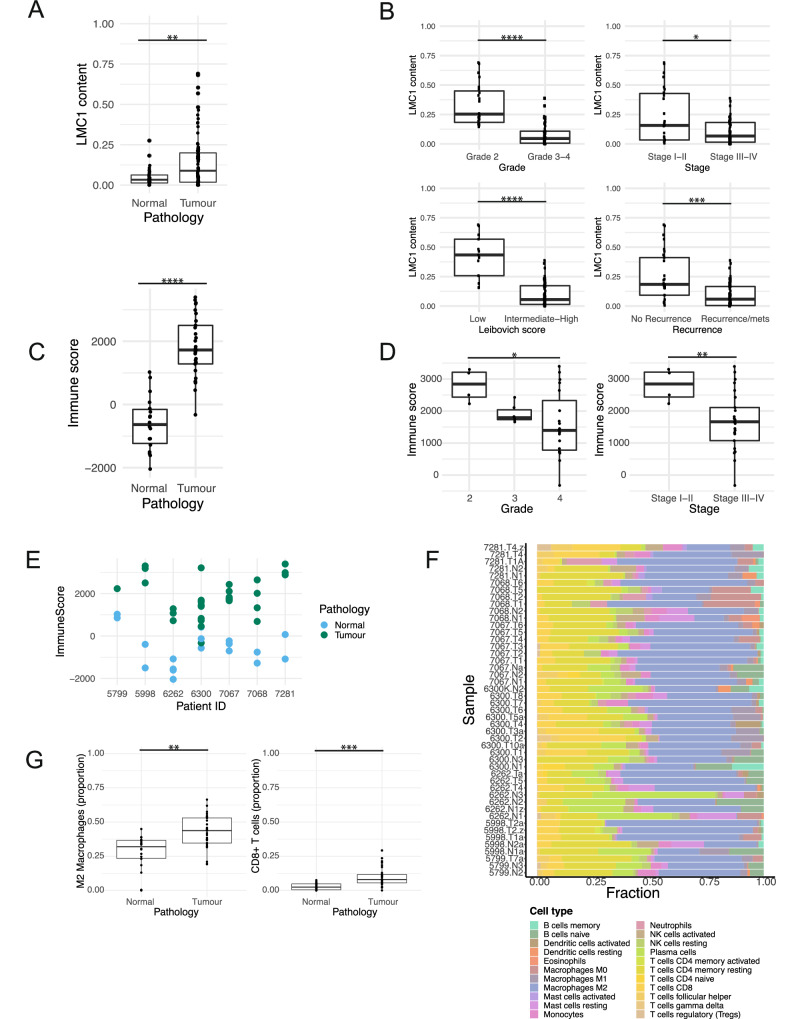
Immune cell components in tumour and normal samples, by clinical parameters. Boxplots depicting the LMC1 (latent methylation component 1) content obtained from methylation deconvolution analysis. LMC1 content is shown in ccRCC vs normal kidney (**A**). The LMC1 component in tumour samples is also shown by grade, stage, Leibovich score and recurrence status (**B**). Boxplots depicting the immune score obtained from RNA-seq analysis (using ‘ESTIMATE’) for tumour vs normal samples (**C**). The immune score is shown in tumour samples, by grade and stage (**D**) and by patient (**E**). Results of immune cell decomposition from RNA-seq analysis (using ‘CIBERTSORTx’), for each patient (**F**), and in tumour versus normal samples (**G**). * = *p* value < 0.05; ** = *p* value < 0.01, *** = *p* value < 0.001, **** = *p* value < 0.0001.

Subsequent analysis focused on characterising the clinical significance of LMC1 and LMC3, which represent immune cell infiltration (all reported *p*-values for BH adjusted Wilcoxon test). The proportion of LMC1 was significantly higher in ccRCC compared to normal kidney samples (median 9% versus 3%, adjusted *p* value = 0.004, Fig. 7A). Interestingly, LMC1 was significantly higher in tumour samples with favourable clinical prognostic parameters (Fig. 7B). LMC1 was significantly higher in low grade disease (25% in grade 2 versus 5% in grades 3–4, adjusted *p* value = 5.5e-09), the absence of necrosis (19% in the presence of necrosis vs 5% in the absence of necrosis, adjusted *p* value = 0.0005), lower stage (16% stage I-II versus 7% versus III-IV, adjusted *p* value = 0.028), lower Leibovich score (43% low risk versus 6% intermediate-high risk, adjusted *p* value = 1.1e-06) and better prognosis (18% no recurrence vs 6% recurrence or metastases at diagnosis, adjusted *p* value = 0.0004) (Fig. 7B and Supplementary Figure S12). The fact that LMC1 was consistently higher for all four clinical parameters suggests this may be relevant and less likely to have been noted by chance. LMC3 was not related to any clinical parameter.

An orthogonal analysis was performed using RNA-seq data to calculate an immune score for each sample, and this was noted to follow a similar pattern as was noted for LMC1. Indeed, the immune score was significantly higher in ccRCC compared to normal kidney (median score 1725 vs -636, adjusted *p* value = 4.0e-10; Wilcoxon test; Fig. 7C). A higher immune score was also associated with more favourable prognostic factors (lower stage and grade, Wilcoxon test adjusted *p* value < 0.05 for all comparisons, Fig. 7D). It was not possible to assess the impact of Leibovich score and recurrence status separately due to small sample sizes. The immune score was also negatively correlated with tumour size (correlation = −0.61, *p* value = 0.0005, correlation test). The immune score was relatively similar in most multi-region tumour samples from the same patient, except for patient 6300 which had higher variability (Fig. 7E). Next, we used ‘CIBERSORTx’ [28] to deconvolute bulk RNA-seq data from our samples to try to explore the underlying immune cell types which may contribute to LMC1 and the immune score pattern noted (Fig. 7F). There was a significantly higher proportion of M2 macrophages and CD8+ T cells in ccRCC compared to normal tissue (median 8% vs 2%, adjusted *p* value = 0.004 and median 44% vs 32%, adjusted *p* value = 0.001 respectively; Wilcoxon test; Fig. 7G). The small sample size and large number of immune cell types precluded an evaluation of cell types by clinical parameters.

## Discussion

We explore ccRCC heterogeneity between patients, within a patient and within a sample (i.e. epipolymorphism). Heterogeneity between patients was dominant over heterogeneity within a patient and this was consistent with the limited available evidence in ccRCC [9–11, 13], colorectal and lung cancer [17, 29]. We compared phylogenetic and phylo-epigenetic trees and demonstrate similarities in evolutionary trajectories in a subset of patients, with differing trajectories in others. This is the first such comprehensive analysis to be performed in ccRCC. Studies in papillary RCC, prostate cancer and glioma suggest that similarities between methylation and SCNA phylogenies are expected and could point towards co-evolution [18, 30].

Our main finding was that differential epipolymorphism was noted between ccRCC and normal tissue, in the promoter region of genes which are known to be associated with kidney cancer. This was identified in a primary cohort of samples (i.e. 135 multi-region samples) and externally validated in a separate cohort of 71 non multi-region samples. These results suggest that disordered methylation may accumulate in functionally relevant loci which are known to contribute to tumorigenesis in ccRCC. Furthermore, we demonstrated that differential epipolymorphism in the gene promoter was a predictor of gene expression, after adjusting for average methylation. Most commonly, there was a negative association, in keeping with transcriptional silencing. In keeping with our findings, Landau et al. previously demonstrated that locally disordered methylation predicts gene expression in chronic lymphocytic leukemia [22], and ours is the first such exploration in ccRCC. Hu et al. evaluated temporally distinct samples along the lung carcinogenesis pathway [31] and demonstrated higher levels of epipolymorphism as cancer progressed from earlier to later stage disease, suggesting that DNA methylation had undergone evolutionary drift. Future research should therefore focus on exploring epipolymorphism in early vs later stage ccRCC to glean important information regarding tumorigenesis.

Furthermore, we identified heterogeneously methylated DMCs which are likely to be subclonal events as they coincide with putative prognostic markers in ccRCC (such as *SFRP1*, *DKK2* and *CCND1*) [32]. These methylation changes may be late events in tumorigenesis and could represent markers of tumour aggressiveness. Heterogeneous methylation within these gene promoters could explain difficulties validating these prognostic markers noted in the literature [32]. We also demonstrate that a proportion of DMCs (i.e. those with a high variance in tumour) may represent immune cell markers rather than tumour intrinsic changes. We performed reference-free deconvolution of bulk methylome data into seven latent methylation components (LMCs). We demonstrate that LMC1 levels, which are likely to represent a T cell infiltrate, were higher in tumours which were lower grade, stage and did not develop a recurrence. In keeping with our results, ccRCC has been found to have a high immune cell infiltrate relative to other cancers, and in particular a high T cell infiltrate [33, 34], and a high number of CD8 + T cells was associated with poor overall survival [35].

A strength of our work is the use of sequence level methylation data using Epic-seq, which allows biological discovery not amenable to array-based methods (such as 850k array). An additional strength is the external validation in an independent cohort of patients and the exploration in cell line data to remove the confounding effect of tumour purity. DNA methylation changes may occur in cell lines secondary to immortalization and growth in-vitro [11, 36], which needs to be taken into consideration when interpreting results. A limitation is that methylation and epipolymorphism are interrelated and thus their effects on gene expression are difficult to disentangle, nor is it possible to demonstrate direct causation. Despite these caveats, these novel results suggest that epipolymorphism within a gene promoter may be an independent regulator of gene expression in addition to overall methylation in ccRCC. Lack of clinical correlates in our study may be due to the relatively small sample size (*N* = 18) or due to limited or incomplete clinical follow-up, with absence of time-to-recurrence data. Alternatively, it may be that the relationship between methylation ITH and prognosis is not linear, as is the case for mutation ITH. Indeed, Turajlic et al. noted that whilst ccRCC patients with low mutational ITH have attenuated progression, patients with both low mutational ITH and high chromosomal instability index have rapid progression [3]. Lastly, although we demonstrate that LMC1 is likely to represent a T cell population, the present work was unable to determine the exact cell type and further external validation is required.

In summary, this analysis represents a systematic characterization of ccRCC methylation heterogeneity and the relationship with gene expression and copy number aberrations. Furthermore, we have conducted the first evaluation of epipolymorphism in ccRCC. We analyse sequence-level data and provide novel insights into methylation epipolymorphism and the functional relevance on gene expression, gleaning important information on tumour biology and biomarker selection. Future studies exploring epipolymorphism in different stages of ccRCC are needed to assess whether there may be there may be evolutionary changes associated with disease progression. Furthermore, further work should focus on characterizing and validating the link between high T cell infiltrate in ccRCC and prognosis.

## Methods

Detailed methods are available in the Supplementary methods.

### Samples

Fresh-frozen normal kidney and multi-region tumour tissue samples were obtained from patients undergoing nephrectomy at Addenbrooke’s Hospital between 2010–2018 (DIAMOND; Ethics REC ID 03/018). DNA and RNA were extracted using the AllPrep DNA/RNA Mini Kit (QIAGEN). DNA was obtained from the 786-O and OS-RC-2 cell lines.

### DNA methylation analysis

Methylation analysis was performed using the Illumina TruSeq Methyl Capture EPIC Library Preparation Kit (Epic-seq) [37]. Sequencing, trimming, alignment, and methylation calling were performed as previously described [37]. CpGs located at the site of C/T and G/A SNPs were removed [38]. DNA methylation age was calculated using Horvath’s epigenetic clock [39] and the predicted to chronological age ratio (PCAR) was calculated by dividing DNA methylation age by age [40]. Phylo-epigenetic trees were created using the top 10% of CpGs with the highest variance in tumour samples using ‘Ape’ v5.5 [41]. DMC analysis was assessed at individual CpGs using ‘methylKit’ v1.12.0 [42]. Publicly available 450k array data were obtained for the TCGA KIRC dataset (160 normal kidney and 325 ccRCC samples) [43].

### Somatic copy number aberration and mutation analysis

WES was performed on multi-region tumour tissue, normal kidney tissue samples and/or germline buffy coat DNA, using the Illumina Nextera™ Flex for Enrichment protocol or as previously described [44]. Sequenced data were aligned to the human reference genome (GRCh38/hg38) using bwa v0.7.17 (bwa-mem algorithm, default settings). SCNA phylogenetic trees were created using ‘Minimum-Event Distance for Intra-tumour Copy-number Comparisons’ (MEDICC2) [45, 46]. The Robinson-Fould measure was used compare similarities between phylogenetic and phylo-epigenetic trees, using ‘TreeDist’ v2.2.0 [47]. Mutation calling was performed using Mutect2 in GATK (default filtering settings) as previously described [48], and all further analysis and data visualisation was performed using maftools [49].

### Analysis of gene expression

RNA-seq was performed using the Illumina TruSeq stranded Total RNA kit. After alignment to the human transcriptome with ‘Salmon’ v1.4.0 [50], transcriptome level count data were converted to gene level data using ‘tximport’ v1.14.2 [51]. Genes with low expression (counts <5) were removed. ‘DESeq2’ v1.32.0 was utilised to evaluate differentially expressed genes in ccRCC versus normal kidney, using non-normalised count level data and patient ID as a covariate (to account for multi-region sampling) [52]. RNA-seq data were also used to determine the ClearCode34 risk score for each sample, as previously described [19, 20].

### DNA methylation epipolymorphism

Epipolymorphism was assessed using ‘methclone’ [12, 53, 54]. E-loci were included in the analysis if methylation data were present in ≥75% of samples, at ≥10x coverage. ‘Epihet’ v1.2.0 was used to identify e-loci with significant differential epipolymorphism (absolute epipolymorphism difference >0.1 and adjusted *p* value < 0.01) in ccRCC versus normal kidney [54]. Results were externally validated in an independent cohort of ccRCC and normal kidney samples evaluated using Epic-seq (*N* = 71 samples) and 786-O cell lines. We evaluated whether average methylation and epipolymorphism may predict gene expression using matched Epic-seq and RNA-seq data (*N* = 47), as previously described [22]. First, we evaluated a linear model predicting gene expression based on epipolymorphism, with a Benjamini–Hochberg correction for multiple testing. To ascertain the effect of epipolymorphism beyond methylation, we evaluated a linear model predicting gene expression based on methylation alone or methylation and epipolymorphism and compared the adjusted *R*^2^ from the two models using a likelihood ratio test [22].

### Purity and deconvolution analysis

Sample purity was evaluated using: DNA methylation using ‘InfiniumPurify’ [55], RNA-seq using ‘ESTIMATE’ [27] and WES using ‘ASCAT’ [56] (Supplementary Figure S11). We performed cell type deconvolution from methylation data using ‘MeDeCom’ v1.0.0 [26, 57], using the top 10% of DMCs with the highest methylation variance in tumour tissue (*N* = 10794 DMCs). The Wilcoxon signed rank sum test was used to assess the difference in the LMC content by tumour stage (stage I-II vs III-IV), grade, Leibovich score (low versus intermediate/high) and recurrence status (no recurrence vs recurrence), whilst adjusting for multiple testing. We performed cell type deconvolution using RNA-seq data and ‘CIBERSORTx’ [28], via ‘Immunedeconv’ v2.0.4 [58].

## Supplementary information

supplementary methods

supplementary figures and tables

## Data Availability

The datasets generated during and/or analysed during the current study will be available in The European Genome-phenome Archive (EGA) repository upon manuscript publication.
