# Supplementary material for: Evidence of DNA methylation heterogeneity and epipolymorphism in kidney cancer tissue samples
Source: Oncogene. 2025 Jan 17;44(15):1024–36. doi: 10.1038/s41388-024-03270-3 (PMC11976292; doi:10.1038/s41388-024-03270-3)
Supplement: Supplementary file 1 — supplementary methods [file 41388_2024_3270_MOESM1_ESM.docx]

# Supplemental methods

Table of Contents

[Supplemental methods 1](#_Toc182486325)

[Samples 2](#_Toc182486326)

[Patient nephrectomy tissue samples 2](#_Toc182486327)

[Cell lines 3](#_Toc182486328)

[Experimental methods 3](#_Toc182486329)

[Nucleic acid extraction from tissue samples 3](#_Toc182486330)

[Epic-seq library preparation and sequencing 4](#_Toc182486331)

[Whole exome library preparation and sequencing 4](#_Toc182486332)

[RNA-seq library preparation and sequencing 5](#_Toc182486333)

[Publicly available datasets 5](#_Toc182486334)

[Data analysis: general methods 6](#_Toc182486335)

[Data processing 6](#_Toc182486336)

[Data visualisation and statistical analysis 7](#_Toc182486337)

[Differential methylation analysis in tissue 7](#_Toc182486338)

[Overlapping Epic-seq and Array datasets 8](#_Toc182486339)

[Evaluating the CpG Island Methylator Phenotype 8](#_Toc182486340)

[Annotation and enrichment analysis 8](#_Toc182486341)

[Gene expression using RNA-seq 9](#_Toc182486342)

[Mutation calling using WES 9](#_Toc182486343)

[Tumour purity assessment and cell type deconvolution 9](#_Toc182486344)

[Purity and deconvolution using DNA methylation data 9](#_Toc182486345)

[Purity and deconvolution using RNA-seq and WES data 10](#_Toc182486346)

[Analysis of methylation heterogeneity in ccRCC tissue 11](#_Toc182486347)

[Heterogeneity between patients 11](#_Toc182486348)

[Heterogeneity within a patient 12](#_Toc182486349)

[Heterogeneity within a sample 14](#_Toc182486350)

## Samples

### Patient nephrectomy tissue samples

A cohort of patients with benign and malignant renal tumours was identified from a biobanking study at Addenbrooke’s Hospital: ‘Discovery and analysis of novel biomarkers in urological diseases’ (DIAMOND; REC ID 03/018). Ethical approval and patient consent were obtained. All participants were assigned anonymous IDs. The patients were included for analysis aiming to represent a spectrum of disease severity, thereby including both patients with small renal masses, patients with locally advanced disease and metastases. For tissue analysis, samples (tumour and adjacent normal) were obtained from patients undergoing curative or cytoreductive nephrectomy between 2010 and 2018. Tissue samples were collected by the pathology team at Addenbrooke’s Hospital (as described below), embedded in OCT (optimal cutting temperature) compound, sectioned and stored at -80°C. Subsequently, we received fresh frozen tissue specimens directly from the Tissue Bank.

In a subset of patients, multi-region tumour samples were collected by the pathologist along with adjacent normal kidney tissue. For samples taken post 2016, ‘true’ multi-region samples were collected from nephrectomy specimens using a 6mm core biopsy puncher and tumour maps delineating the location of multi-region sampling were available. For samples prior to 2016, multiple slices of renal tissue were obtained using a scalpel, however unfortunately maps were not available. In addition, several patients had large tissue slices that were subdivided into samples ‘a’ and ‘b’ representing distinct tissue samples from the same area of the kidney, but only a few millimetres apart. This is useful as it enables us to assess the similarity of samples which are spatially very close and are therefore expected to be similar.

#### Clinical data

Clinical annotation, including patient details and tumour pathological characteristics, was performed by retrospective review of prospectively maintained hospital records. Unfortunately, clinical data were only available for a subset of patients included in the analysis at the time of writing (although further data have been requested). The following parameters were obtained from the nephrectomy pathology report: tumour size (maximal diameter in cm), Fuhrman grade, pathological stage and presence of necrosis. Patient details included age, sex and body mass index (BMI) at the time of sampling. Leibovich score and recurrence data were available for a subset of patients (≥4 years follow up for every patient). Recurrence was defined as local relapse at the nephrectomy site or new metastases on CT imaging on clinical follow up.

### Cell lines

Genomic DNA (gDNA) was collected from the 786-O cell line to represent a model system of ccRCC. 786-O is one of the top three most cited and most well characterised RCC cell lines. The cell line was originally derived from a 58 year old Caucasian man with primary ccRCC and widespread metastases, and is characterised by a homozygous mutation in the VHL gene [1]. gDNA from 786-O cells was provided by Dr Paulo Rodrigues (Vanharanta Group, Hutchison MRC Institute). 786-O cells were cultured in DMEM/F12 supplemented with B27 (Invitrogen), streptomycin (μg/ml), and EGF/FGF (Peprotech 10 ng/mL). DNA from OS-RC-2 cell lines was kindly provided by Paulo Rodrigues (Vanharanta laboratory) and cultured as previously described [2]. Cell lines were authenticated using short-tandem repeat genetic profiling. The CRUK CI Cell Services Core Facility performed genotyping and data analysis using the Applied Biosystems Gene Mapper 5 software. The percentage match to the reference profile was 100%.

## Experimental methods

###

### Nucleic acid extraction from tissue samples

Nucleic acid extraction from tissue was performed in one of two ways. For some cases, gDNA was extracted from a small section of frozen tissue (approximately 20mg), using the commercially available DNeasy Blood & Tissue kit (QIAGEN) according to the manufacturers protocol (therefore RNA not available). For the remainder of patients, DNA and RNA were extracted using the AllPrep DNA/RNA Mini Kit (QIAGEN) according to the manufacturers protocol. We quantified DNA using the Qubit^TM^ 4 fluorometer (ThermoFisher Scientific). Where gDNA concentration was below the required threshold for subsequent analysis (<9ng/µl), the SpeedVac Vacuum Concentrator (ThermoFisher Scientific) was used to increase DNA concentration.

### Epic-seq library preparation and sequencing

We used the TruSeq Methyl Capture EPIC Library Preparation Kit (Illumina), hereby referred to as Epic-seq, to evaluate methylation in gDNA obtained from tissue. We sheared gDNA samples (10ng/µl, 500ng total) using the S220 Focused-ultrasonicator (Covaris) to generate dsDNA fragments. Samples were sheared for 280 seconds using the following shearing settings: 175W peak incident power, 10% duty factor, 200 cycles per burst. The D1000 ScreenTape System (Agilent) was used to ensure >60% of DNA fragments were between 100 and 300bp long, with a mean fragment size of 180-200bp. The Epic-seq library preparation was performed using the manufacturers protocol. This consists of a capture-based method targeting ~3 million CpGs. Four samples were multiplexed in each capture reaction using sample indexing adaptors. The protocol involves hybridization of biotin-tagged probes to gDNA followed by capture using streptavidin beads (two hybridization-capture steps) followed by bisulphite conversion at 54°C for two hours. Twelve samples were pooled for sequencing on the HiSeq4000 Illumina Sequencing platform (single end 150bp read; 20% PhiX) using two lanes per library pool. We performed two technical replicates for cell line data (gDNA derived from HK2 cell lines) and evaluated CpG methylation. We focused the analysis on CpGs which achieved ≥10x minimum coverage and demonstrated that the Pearson correlation coefficient between technical replicates was 0.97, suggesting assay reproducibility.

### Whole exome library preparation and sequencing

In a subset of patients with ccRCC used for methylation analysis, whole exome sequencing (WES) of multi-region tumour tissue, normal kidney tissue samples and/or germline buffy coat DNA had previously been performed by Dr Christopher Smith at the Rosenfeld Group, CRUK CI, as previously described [3]. In brief, 50ng of gDNA were fragmented using the S220 Focused-ultrasonicator (Covaris). Library preparation was performed using the Thruplex DNA-Seq protocol (Rubicon Genomics; 5 PCR cycles). Next, exomes were captured using the TruSeq Exome Capture protocol (Illumina) according to the manufacturers protocol. Libraries were amplified (8 PCR cycles) and subsequently sequenced on the Hiseq4000 platform (Illumina). To increase the total number of samples available for the analysis, WES was performed for an additional set of samples. WES was undertaken by the genomics core at the CRUK CI, using Nextera™ Flex for Enrichment protocol (Illumina) according to the manufacturers protocol. Sequencing was performed using NovaSeq (paired end, 150bp, 55 samples on two lanes of S4).

### RNA-seq library preparation and sequencing

RNA was available for a subset of multi-region samples (N=47) from patients with ccRCC used for methylation and WES analysis. We evaluated RNA integrity (RIN), measured on a scale from 0 to 10, using the Tapestation (Agilent). Unfortunately, the RNA was low quality (RIN values: median 5.8, minimum 1.4, maximum 8.8). Delays in sample freezing, processing and freeze/thaw cycles may have contributed to RNA degradation. RNA-seq was performed using the Illumina TruSeq stranded Total RNA kit on 225ng of RNA, according to the manufacturers protocol, by the genomics core at the CRUK CI. For library preparation, the location of the tumour and normal samples was randomised on the plate to remove variability/batch effect related to the plate. In brief, ribosomal RNA (rRNA) was depleted using biotinylated, target-specific oligos combined with Ribo-Zero rRNA removal beads, to ensure only messenger RNA (mRNA) was left. Following this, the samples were fragmented for 2 minutes (due to the relatively low quality RNA). RNA was then copied to DNA using reverse transcriptase, the library was prepared and amplified (15 PCR cycles). Libraries were sequenced using NovaSeq (paired end, 50bp, 47 samples on 3 lanes of Novaseq SP) aiming to achieve approximately 10 million reads per sample.

## Publicly available datasets

TCGA data for kidney cancer tissue samples (ccRCC and adjacent normal) were obtained via the ‘TCGAbiolinks’ package v2.20.1 in R [4]. In TCGA, methylation data were assessed using the Illumina Infinium Human DNA Methylation 450k platform (450k array). Pre-processed beta values were downloaded for samples, along with clinical and sample characteristics. TCGA data were also evaluated using the following online interactive tools: ‘TCGA Wanderer’ [5] and ‘cBioPortal’ [6]. For deconvolution analysis, we obtained reference methylomes for various cell types (Table 1) from GEO and the ENCODE project [7]. In order to obtain reference methylomes for kidney cell lines, we performed Epic-seq on HK2 and 786-O cells. Some of the publicly available datasets used previous genome builds (as shown in Table 1), therefore we converted these to hg38 using the ‘liftover’ function in the ‘rtracklayer’ package v1.52.1 in R [8].

| Table 1: Reference methylomes used for methylation deconvolution analysis | | | |
| --- | --- | --- | --- |
| Data | Sample type | Data type/ Platform | Source |
| Salas et al  [9] | Immune cells | 850k array | GEO accession number: GSE110555  Sample identifiers:  GSM2998022 = NK cell  GSM2998024 = B cell  GSM2998030 = neutrophil  GSM2998032 = CD8+ T cell  GSM2998039 = monocyte  GSM2998048 = CD4+ T cell |
| ENCODE [7] | Immune cells | WGBS (hg38)  Minimum coverage 5x | ENCODE project, sample identifiers:  ENCFF649RBS = myeloid progenitor  ENCFF689TNG = monocyte  ENCFF703XLD = B cell  ENCFF953DKC = T cell |
| ENCODE [7] | Adipose cell | WGBS (hg38)  Minimum coverage 5x | ENCODE project, sample identifiers:  ENCFF528JMA = adipose cell |
| ENCODE [7] | Skin fibroblast  (Primary cell) | WGBS (hg38)  Minimum coverage 5x | ENCODE project, sample identifiers:  GM23248  ENCFF752NXS = fibroblast replicate 1  ENCFF116DGM= fibroblast replicate2 |
| Generated by me | HK2 & 786-O cell lines | Epic-seq (hg38)  Minimum coverage 10x | Data generated by the study authors |

Abbreviations: WGBS = whole genome bisulphite sequencing

##

## Data analysis: general methods

### Data processing

For methylation, sequencing data were processed by Sara Pita (research assistant, Massie Group) using an in-house pipeline. Sequenced data were trimmed (TrimGalore v0.4.4) and aligned to the bisulphite converted human reference genome (GRCh38/hg38) using Bismark (v0.22.1) and Bowtie2 (v2.4). For gDNA derived from tissue and prepared with Epic-seq, duplicate reads were maintained. We evaluated quality control metrics using Picard Tools (default settings) [10] and the output from MultiQC.

For RNA-seq and WES, data processing was performed by Kamal Kishore at the bioinformatics core at the CRUK CI. For WES, sequenced data were aligned to the human reference genome (GRCh38/hg38) using bwa v0.7.17 (bwa-mem algorithm, using default settings). For RNA-seq, the results were aligned to the reference transcriptome using ‘Salmon’ v1.4.0 [11].

### Data visualisation and statistical analysis

Data visualisation and analysis was performed using the R statistical software (version 3.6.1 subsequently upgraded to 4.1.1). We created figure schematics using Biorender (Biorender.com). Unsupervised clustering was performed using principal component analysis (PCA) and/or dendrograms. Heatmaps were used to depict DNA methylation and gene expression for multiple samples, using the ‘ComplexHeatmap’ package v2.8.0 [12]. In addition, locus plots were created to visualise DNA methylation levels for each sample, at each CpG within a given locus (reference genome hg38).

Group differences were compared using Fisher’s exact test (for categorical variables), Mann-Whitney or Wilcoxon Signed Rank Sum test (for non-parametric data) and Student’s t test (for parametric data). For continuous variables, association was evaluated using Pearson’s correlation coefficient and/or linear regression models to obtain an adjusted R^2^, along with p values. P values were corrected for multiple testing using a Benjamini–Hochberg correction.

### Differential methylation analysis in tissue

We generated gDNA methylation data using Epic-seq. The percentage methylation at a given locus was obtained by counting the number of methylated cytosines divided by the total number of reads (i.e. number of Cs/number of Ts+Cs). Methylation levels therefore range between 0 and 1, and this is often referred to as the beta value. CpGs located on the sex chromosomes were omitted to remove gender bias. In addition, CpGs located at the site of C/T and G/A SNPs were removed as these cannot be distinguished from differential DNA methylation in single read data [13]. Data were included in downstream analyses if a depth of ≥10x coverage was achieved, to reduce the risk of false positive calling. This level of minimum coverage allows a 10% methylation difference to be called (i.e. 1 out of 10 reads). Data were therefore available for 2.9 million CpGs.

We performed differentially methylated cytosine (DMC) analysis at individual CpGs using the ‘methylKit’ package v1.12.0 in R [14]. This package uses logistic regression to compare CpGs between two groups (e.g. tumour *vs* normal), with p-value adjustment for multiple testing. Patient ID was used as a covariate in logistic regression to adjust for multi-region sampling. Significant DMCs were defined as ≥25% absolute methylation difference between groups (a commonly used cut-point in the literature) and q value <0.01 [14].

### Overlapping Epic-seq and Array datasets

The Epic-seq method generates sequence level data on approximately 3 million CpGs; in contrast with the approximately 27,000 CpGs included in the Illumina 27k array, 450,000 CpGs included in the Illumina 450k array and 850,000 in the Illumina 850k array. A number of analyses required an external validation set from publicly available sources (e.g. TCGA) or conversion of publicly available methylomes using 450k or 850k arrays (for example for deconvolution analysis). In order to combine data from the two methods, Epic-seq methylation values within ±50bp of the 450k probes were averaged, as adjacent CpGs tend to be co-methylated [15]. This has previously been demonstrated to be a valid approach; 8 technical replicates (fresh frozen tissue) assessed on both Illumina Epic-seq and the 450k array obtained a correlation ≥0.96 using this method. After combining datasets, we removed CpG probes found in two blacklists [16, 17] on the 450k array. These consist of CpG probes that either map to multiple regions, are located at repeat regions, on sex chromosomes or at the site of C/T and G/A SNPs.

### Evaluating the CpG Island Methylator Phenotype

Arai et al identified 16 CpGs which are hypermethylated and characteristic of the aggressive CpG island methylator phenotype (CIMP) [18]. Methylation values were available for 13 of these CpGs in our dataset and were used to cluster tumour samples (using a dendrogram) in order to explore the CIMP phenotype. In addition, we evaluated CIMP as previously described [19]. We performed an unsupervised hierarchical clustering using Ward’s D2 method on the top 343 CpGs based on standard deviation in tumour samples that are unmethylated in normal samples (mean beta value <0.1). The clustering dendrogram was cut to k=3.

### Annotation and enrichment analysis

We annotated CpGs of interest to the human reference genome (hg38) to determine their location relative to CpG islands and shores, as well as annotating to the nearest proximal gene using the ‘methylKit’ v1.12.0 and ‘ChIPseeker’ v1.22.1 packages respectively [14, 20]. The hg38 annotation source was obtained from the ‘EnsDb.Hsapiens.v86’ package v2.99.0 in R [21]. CpGs which were within 1.5kb of the promoter region were then selected for enrichment analysis. Gene set enrichment analysis and ontology (including disease ontology, biological processes, molecular functions) were performed using ‘clusterProfiler’ v3.14.3 in R [22], where the background set was defined as the total number of features evaluated.

### Gene expression using RNA-seq

RNA-seq data were generated for 47 fresh-frozen ccRCC and normal kidney tissue samples. After alignment to the human transcriptome with ‘Salmon’ v1.4.0 [11], we converted transcriptome level count data to gene level data using ‘tximport’ v1.14.2 in R [23]. Genes with low expression (counts <5) were subsequently removed. ‘DESeq2’ v1.32.0 was utilised to evaluate differentially expressed genes in ccRCC versus normal kidney, using non-normalised count level data [24]. Importantly, Patient ID was used as a covariate to account for multi-region sampling, and p values were adjusted for multiple testing. RNA-seq data were also used to determine the ClearCode34 prognostic risk score for each sample, as described by the study authors [25, 26]. In brief, data were available for 31 out of the 34 ClearCode34 genes. First data were median centred, then log transformed (log2 + 0.1) and visualised in a heatmap with unsupervised hierarchical clustering.

### Mutation calling using WES

Mutation calling was performed using Mutect2 in GATK, developed by the Broad Institute (default filtering settings), as previously described [27]. Annotation was performed using Functotator for all PASS filtered variants. All further analysis and data visualisation was performed using maftools [28].

## Tumour purity assessment and cell type deconvolution

### Purity and deconvolution using DNA methylation data

We estimated tumour purity from methylation data using the ‘InfiniumPurify’ package v1.3.1 in R, as previously described [29]. Importantly, the package estimates tumour purity relative to contamination with normal (non-cancerous) tissue. The function compares methylation in normal and tumour tissue (taking into account the variance of methylation in tumour) and identifies informative differentially methylated CpG sites (iDMCs). iDMCs are then used to estimate purity using Gaussian kernel density [29].

Percentage methylation at CpGs for each sample (i.e. beta values) represent a mixture of methylation values from reads derived from different cell types. We performed cell type deconvolution from methylation data using the ‘MeDeCom’ package v1.0.0 in R [30, 31]. Good quality reference epigenome maps for ccRCC purified cell components are lacking (lack of available data and poor overlap between CpGs covered by Epic-seq). Therefore, we selected a ‘reference-free’ deconvolution method to perform an unconstrained analysis. First, feature selection was performed: DMCs were ranked based on the highest methylation variance in tumour tissue and the top 10% (N=10794 DMCs) were identified in order to select the most informative features. ‘MeDeCom’ is unable to handle missing data, therefore imputation was performed for missing CpGs using the k nearest neighbour (‘impute’ package v1.60.0 in R) [32]. ‘MeDeCom’ uses regularized non-negative matrix factorization to decompose the DNA methylation matrix into two matrices: cell-type-specific latent methylation components (LMCs) and the proportion of LMCs in each sample [31]. LMCs represent the reference methylomes of unknown cell populations. The method was run over multiple iterations and the parameters *K* (i.e., the number of LMCs) and *λ* (i.e., regularization parameter) which minimize the cross-validation error were selected. In order to identify the potential cell type corresponding to each LMC, these were correlated with reference methylomes for known cell types. We performed Epic-seq to establish reference methylomes for the HK2 and 786-O cell lines, representing normal kidney proximal epithelium and ccRCC tumour respectively. The remainder of the cell type reference methylomes were obtained from the literature. The LMCs were also correlated with purity estimates derived by ‘InfiniumPurify’ (DNA methylation), ‘ESTIMATE’ (RNA-seq) and ‘ASCAT’ (WES). The Wilcoxon signed rank sum test was used to assess whether there was a significant difference in the LMC content by tumour stage (stage I-II vs III-IV), grade, Leibovich score (low versus intermediate/high) and recurrence status (no recurrence vs recurrence), whilst adjusting for multiple testing.

### Purity and deconvolution using RNA-seq and WES data

We used RNA-seq data to estimate tumour purity using the ‘Estimation of STromal and Immune cells in MAlignant Tumours using Expression data’ (ESTIMATE) package v1.0.13 in R, as previously described [33]. ‘ESTIMATE’ determines tumour purity as a function of admixtures of immune and stromal cell components based on gene expression data. We compared immune and stromal cell components derived from ‘ESTIMATE’ for our dataset (N=47) and TCGA data, confirming our results were within the expected range. Tumour purity was estimated from WES data using ‘ASCAT’ [34]. Subsequently, Pearson’s correlation coefficient was used to compare the association between purity calculated using WES, RNA-seq and Epic-seq.

In addition, we performed cell type deconvolution using RNA-seq data and ‘CIBERSORTx’ [35], via the ‘Immunedeconv’ package v2.0.4 in R [36]. Gene expression data were normalised (normalization method: transcripts per million, not log transformed) prior to decomposition. ‘CIBERSORTx’ enables the deconvolution of bulk RNA-seq data into 22 immune cell types, based on a reference transcriptome provided by the study authors (termed the ‘LM22 signature matrix’) [35].

##

## Analysis of methylation heterogeneity in ccRCC tissue

We performed an analysis of methylation heterogeneity on three levels: between patients, within a patient and within a sample, as previously described [37]. We performed all the methylation analyses using Epic-seq data generated from ccRCC and normal kidney tissue.

### Heterogeneity between patients

Heterogeneity between patients was defined as an evaluation of methylation patterns in tumour samples from different patients with the same diagnosis (i.e. ccRCC). In order to evaluate methylation heterogeneity between patients, methylation beta values were obtained. All CpGs at SNP sites were removed (i.e. not just C/T and G/A SNPs) to ensure that the heterogeneity noted between patients was driven by methylation rather than SNPs. Principal component analysis was performed using data from all available CpGs (~1.1million CpGs) and sample clustering was evaluated. Subsequently the top-most variable CpG (i.e. CpGs with the highest variance in tumour samples) were selected for visualisation in a heatmap. This analysis was compared for the top 10,000 CpGs and the top 50,000 CpGs.

### Heterogeneity within a patient

Heterogeneity within a patient was defined as an evaluation of methylation patterns amongst multi-region tumour samples obtained from one patient (i.e. multiple tumour and normal samples are taken from each individual ccRCC tumour). ITH was evaluated by assessing epigenetic age and phylogenetic trees derived from methylation and somatic copy number data (SCNA). For each of these parameters, we evaluated the association with clinical and prognostic factors. We assessed whether there was a significant difference by tumour stage (stage I-II vs III-IV), grade, tumour size, Leibovich score (low vs intermediate/high) and recurrence status (no recurrence vs recurrence), whilst adjusting for multiple testing.

#### DNA methylation age

The predicted DNA methylation age of each sample was calculated using Horvath’s epigenetic clock, using publicly available code published by the study authors [38]. In order to demonstrate reliability of methods, first we evaluated methylation age for TCGA ccRCC tumour (N=325) and normal kidney (N=160) tissue samples. Subsequently, we repeated the analysis for my Epic-seq samples. Since Horvath’s clock was developed using the 21k Illumina methylation array, the Epic-seq methylation data for our samples was overlapped with the 21k array by averaging methylation levels within ±100bp of Illumina CpG probes, as described in section 0. The 100bp threshold (rather than 50bp) was selected to increase the number of CpG probes for which data might be available. Epic-seq and TCGA data were combined into one data frame and for missing values, imputation was performed using k-nearest neighbours with the ‘impute’ package v1.60.0 in R (k=10, rowmax=0.25) [32]. The association between chronological age and predicted DNA methylation was evaluated for normal and tumour samples separately, using Pearson’s correlation coefficient. The predicted to chronological age ratio (PCAR) was calculated by dividing DNA methylation age by real age, as previously described [39]. Accelerated ageing was defined as a PCAR ≥ 1.

#### Average Pairwise ITH Index (APITH)

Heterogeneity within a patient was evaluated by calculating the Average Pairwise ITH (APITH), using DNA methylation and copy number data respectively, as previously described [40]. For DNA methylation, we calculated the APITH for all CpGs as well as the top 5000 most variable CpGs, using the equation below [40]. Methylation beta values were obtained for tumour samples at the CpGs of interest, subsequently, the pairwise Euclidean distance was calculated and then the average was obtained. In the equation, k represents the total number of samples, and dij is defined as the pairwise Euclidean distance between two samples (i and j):

$$APITH= \frac{2}{k\left( k-1 \right)} \sum_{1\leq i<j\leq k} d_{ij}$$

Subsequently, we explored whether the methylation APITH index may be confounded by tumour purity. Tumour purity was obtained using WES or RNA-seq. We assessed the correlation between the APITH and the variance of the purity of tumour samples derived from the same patient (the latter represents a measure of the spread of purity values). In addition, methylation beta values were adjusted for purity using the ‘Infiniumpurify’ package in R [29], and the methylation APITH score were calculated once again. We compared the APITH index derived from methylation data which were unadjusted versus adjusted for tumour purity, to assess whether this produced similar results.

The copy number APITH index was calculated by Dr Roland Schwarz (Max Delbrück Center in Berlin) by evaluating the percentage of the genome which is affected by private SCNA (using the ‘ASCAT’ package [34]) in each sample and the average pairwise distance between samples, as previously described [40]. We evaluated the correlation between the methylation APITH and the copy number APITH in my dataset using Pearson’s correlation coefficient.

#### Phylogenies using DNA methylation and copy number data

Phylogenies were created using methylation data and SCNA data respectively. Patients were included in the analysis if matched methylation and SCNA data were available on ≥4 tumour samples (N=8 patients), to allow comparisons between phylogenetic and phylo-epigenetic tree topologies. We created phylo-epigenetic trees using the ‘Ape’ package v5.5, where DNA methylation beta values were treated as a continuous variable between 0 and 1 [41]. We selected the top 10% of CpGs with the highest variance in tumour samples and calculated the Euclidean distance matrix. Trees were subsequently inferred using the ordinary least squares minimum evolution algorithm [42], as previously described [43, 44]. Phylogenetic trees were created using SCNA data from WES using ‘Minimum-Event Distance for Intra-tumour Copy-number Comparisons’ (MEDICC2) [45, 46]. In brief, allele-specific copy number analysis was performed using ‘ASCAT’ [34], and subsequently these underwent reference phasing using ‘Refphase’ [47]. ‘MEDICC2’ calculates the pairwise minimum-event distance between samples, and these data are used to create phylogenetic trees using the neighbour joining algorithm [48]. We used the Robinson-Fould measure to compare similarities between phylogenetic and phylo-epigenetic trees for each patient, using the ‘TreeDist’ package v2.2.0 [49]. In brief, the Robinson-Fould measure is derived by counting the number of unique splits which occur in each tree (i.e. splits which occur in one tree and not the other), and the overall metric is normalised (0 to 1 scale) to enable comparisons across trees. In this case a split is defined as a bipartition in a tree which separates two taxa. Phylogenetic and phylo-epigenetic trees were visualised using the ‘plot’ function in the ‘Ape’ package v5.5.

###

### Heterogeneity within a sample

Methylation heterogeneity within a sample was assessed by calculating epipolymorphism. Epipolymorphism is defined as ‘the probability that two epialleles randomly sampled from the locus differ from each other’, where an epigenetic locus (e-locus) consists of four adjacent CpGs in a single sequencing read (i.e. a 150bp window) [50, 51]. Given 4 adjacent CpGs, there are 16 (i.e. 2^4^) possible combinations of methylated and unmethylated cytosines, so there are 16 possible epialleles. Epipolymorphism values were calculated using the ‘methclone’ package [50], using the formula below [52]. In brief, the proportion of each methylation pattern (p) is squared, then all values are summed and subtracted from one. There are a total of 16 possible methylation patterns (i.e. epialleles) therefore in this case S=16.

$$Epipolymorphism= 1- \sum_{i=1}^{S} p_{i}^{2}$$

Epipolymorphism values range between 0 (i.e., fully concordant methylation pattern) and approaching 1 (i.e. highest degree of heterogeneity) [51]. The following section justifies the thresholds/variables used in our analysis. Li et al previously explored the number of adjacent CpGs (up to 10 CpGs) used to define an e-locus and 4 CpGs was selected as this optimised the number of reads and epialleles [50]. Therefore, in our analysis we defined an e-locus as four adjacent CpGs in a single sequencing read. Next, we evaluated the number of e-loci obtained at differing thresholds of minimum coverage. For example, ≥10x coverage obtained 138,412 e-loci, whereas ≥20x coverage obtained 59,480 e-loci, meaning <50% of data compared to ≥10x. Therefore, e-loci were included in my analysis if methylation data were present in ≥75% of samples, at ≥10x coverage in order to increase the number of e-loci that were considered. E-loci located on sex chromosomes were excluded from the analysis. The ‘epihet’ package v1.2.0 was used to compare average epipolymorphism at each e-locus in two groups (for example ccRCC versus normal kidney) and to determine e-loci with significant differential epipolymorphism (defined as absolute epipolymorphism difference >0.1 and adjusted p value <0.01) [52]. The epipolymorphism difference cut-off of 0.1 represents a >10% difference in epipolymorphism values, and is the cut-off routinely used in the literature [52]. The following illustrative example puts this into context. Given 10 reads, if all reads had the same methylation pattern the epipolymorphism would be 0, whereas if one read had a different pattern, then epipolymorphism would be 0.18. Using the selected cut-off of >0.1, this difference would be called as significant (provided the adjusted p value was <0.01).

We evaluated epipolymorphism in a cohort of ccRCC versus normal kidney samples (N=135 samples) to identify e-loci with significant differential epipolymorphism. Significant e-loci were annotated to the nearest gene and GSEA was performed. We externally validated results by assessing differential epipolymorphism in an independent cohort of ccRCC and normal kidney samples (N=71 samples, Epic-seq generated by our team). Subsequently, epipolymorphism was evaluated in 786-O cell lines (4 technical replicates), which represent a model system of ccRCC. Cell lines are 100% pure, thus enabling an evaluation of epipolymorphism which is not confounded by the presence of heterogeneous groups of cells (i.e. gDNA derived from cell lines represents a single cell type, whereas kidney tissue contains multiple cell types).

Average methylation was calculated at each e-locus using the ‘epihet’ package v1.2.0, by calculating the average methylation across 4 adjacent CpGs, across all reads at that locus. Significance was defined as absolute methylation difference >15%, and an adjusted p value < 0.01. Whilst for individual CpGs the commonly used threshold for differential methylation is >25%, a lower threshold was used for average methylation across a read as this includes four adjacent CpGs. The relationship between average methylation and epipolymorphism at each e-locus was evaluated graphically using a scatterplot. Furthermore, we evaluated whether average methylation and epipolymorphism may predict gene expression, as previously described [53]. Matched Epic-seq and RNA-seq data were obtained for a subset of ccRCC and normal kidney samples (N=47). First, we evaluated a linear model predicting gene expression based on epipolymorphism, with a Benjamini-Hochberg (BH) correction for multiple testing. To ascertain the effect of epipolymorphism beyond methylation, we evaluated a linear model predicting gene expression based on methylation alone or methylation and epipolymorphism and compared the adjusted R^2^ from the two models using a likelihood ratio test. The analysis was performed for individual e-loci, and where multiple e-loci were significant for one gene, the e-locus with the lowest BH adjusted p values was shown, along with the number of e-loci per gene. An adjusted p value of <0.05 was considered significant.

**References**

1. Williams RD, Elliott AY, Stein N, Fraley EE. In vitro cultivation of human renal cell cancer. II. Characterization of cell lines. In Vitro. 1978;14(9):779-86.

2. Vanharanta S, Shu W, Brenet F, Hakimi AA, et al. Epigenetic expansion of VHL-HIF signal output drives multiorgan metastasis in renal cancer. Nat Med. 2013;19(1):50-6.

3. Smith CG, Moser T, Mouliere F, Field-Rayner J, et al. Comprehensive characterization of cell-free tumor DNA in plasma and urine of patients with renal tumors. Genome Med. 2020;12(1):23.

4. Colaprico A, Silva TC, Olsen C, Garofano L, et al. TCGAbiolinks: an R/Bioconductor package for integrative analysis of TCGA data. Nucleic Acids Res. 2016;44(8):e71.

5. Diez-Villanueva A, Mallona I, Peinado MA. Wanderer, an interactive viewer to explore DNA methylation and gene expression data in human cancer. Epigenetics Chromatin. 2015;8:22.

6. Cerami E, Gao J, Dogrusoz U, Gross BE, et al. The cBio cancer genomics portal: an open platform for exploring multidimensional cancer genomics data. Cancer Discov. 2012;2(5):401-4.

7. Davis CA, Hitz BC, Sloan CA, Chan ET, et al. The Encyclopedia of DNA elements (ENCODE): data portal update. Nucleic Acids Res. 2018;46(D1):D794-D801.

8. Lawrence M, Gentleman R, Carey V. rtracklayer: an R package for interfacing with genome browsers. Bioinformatics. 2009;25(14):1841-2.

9. Salas LA, Koestler DC, Butler RA, Hansen HM, et al. An optimized library for reference-based deconvolution of whole-blood biospecimens assayed using the Illumina HumanMethylationEPIC BeadArray. Genome biology. 2018;19(1):64.

10. Picard Tools. Broad Institute, GitHub repository [Available from: <http://broadinstitute.github.io/picard/>.

11. Patro R, Duggal G, Love MI, Irizarry RA, Kingsford C. Salmon provides fast and bias-aware quantification of transcript expression. Nat Methods. 2017;14(4):417-9.

12. Gu Z, Eils R, Schlesner M. Complex heatmaps reveal patterns and correlations in multidimensional genomic data. Bioinformatics. 2016;32(18):2847-9.

13. National centre for biotechnology information single nucleotide polymorphisms Homo sapiens HG38 National centre for biotechnology information; 2021 [Available from: <https://ftp.ncbi.nlm.nih.gov/snp/organisms/human_9606_b151_GRCh38p7/VCF/00-common_all.vcf.gz>.

14. Akalin A, Kormaksson M, Li S, Garrett-Bakelman FE, et al. methylKit: a comprehensive R package for the analysis of genome-wide DNA methylation profiles. Genome biology. 2012;13(10):R87.

15. Guo S, Diep D, Plongthongkum N, Fung HL, et al. Identification of methylation haplotype blocks aids in deconvolution of heterogeneous tissue samples and tumor tissue-of-origin mapping from plasma DNA. Nature genetics. 2017;49(4):635-42.

16. Naeem H, Wong NC, Chatterton Z, Hong MK, et al. Reducing the risk of false discovery enabling identification of biologically significant genome-wide methylation status using the HumanMethylation450 array. BMC Genomics. 2014;15:51.

17. Price ME, Cotton AM, Lam LL, Farre P, et al. Additional annotation enhances potential for biologically-relevant analysis of the Illumina Infinium HumanMethylation450 BeadChip array. Epigenetics Chromatin. 2013;6(1):4.

18. Arai E, Chiku S, Mori T, Gotoh M, et al. Single-CpG-resolution methylome analysis identifies clinicopathologically aggressive CpG island methylator phenotype clear cell renal cell carcinomas. Carcinogenesis. 2012;33(8):1487-93.

19. Cancer Genome Atlas Research N, Linehan WM, Spellman PT, Ricketts CJ, et al. Comprehensive Molecular Characterization of Papillary Renal-Cell Carcinoma. The New England journal of medicine. 2016;374(2):135-45.

20. Yu G, Wang LG, He QY. ChIPseeker: an R/Bioconductor package for ChIP peak annotation, comparison and visualization. Bioinformatics. 2015;31(14):2382-3.

21. Rainer J, Gatto L, Weichenberger CX. ensembldb: an R package to create and use Ensembl-based annotation resources. Bioinformatics. 2019;35(17):3151-3.

22. Yu G, Wang LG, Han Y, He QY. clusterProfiler: an R package for comparing biological themes among gene clusters. OMICS. 2012;16(5):284-7.

23. Soneson C, Love MI, Robinson MD. Differential analyses for RNA-seq: transcript-level estimates improve gene-level inferences. F1000Res. 2015;4:1521.

24. Love MI, Huber W, Anders S. Moderated estimation of fold change and dispersion for RNA-seq data with DESeq2. Genome biology. 2014;15(12):550.

25. Brooks SA, Brannon AR, Parker JS, Fisher JC, et al. ClearCode34: A prognostic risk predictor for localized clear cell renal cell carcinoma. Eur Urol. 2014;66(1):77-84.

26. Ghatalia P, Rathmell WK. Systematic Review: ClearCode 34 - A Validated Prognostic Signature in Clear Cell Renal Cell Carcinoma (ccRCC). Kidney Cancer. 2018;2(1):23-9.

27. Cibulskis K, Lawrence MS, Carter SL, Sivachenko A, et al. Sensitive detection of somatic point mutations in impure and heterogeneous cancer samples. Nat Biotechnol. 2013;31(3):213-9.

28. Mayakonda A, Lin DC, Assenov Y, Plass C, Koeffler HP. Maftools: efficient and comprehensive analysis of somatic variants in cancer. Genome Res. 2018;28(11):1747-56.

29. Qin Y, Feng H, Chen M, Wu H, Zheng X. InfiniumPurify: An R package for estimating and accounting for tumor purity in cancer methylation research. Genes Dis. 2018;5(1):43-5.

30. Scherer M, Nazarov PV, Toth R, Sahay S, et al. Reference-free deconvolution, visualization and interpretation of complex DNA methylation data using DecompPipeline, MeDeCom and FactorViz. Nat Protoc. 2020;15(10):3240-63.

31. Lutsik P, Slawski M, Gasparoni G, Vedeneev N, et al. MeDeCom: discovery and quantification of latent components of heterogeneous methylomes. Genome biology. 2017;18(1):55.

32. Hastie T, Tibshirani R, Balasubramanian N, Chu G. impute: impute: Imputation for microarray data. R package version 1.66.0. 2021 [

33. Yoshihara K, Shahmoradgoli M, Martinez E, Vegesna R, et al. Inferring tumour purity and stromal and immune cell admixture from expression data. Nat Commun. 2013;4:2612.

34. Van Loo P, Nordgard SH, Lingjaerde OC, Russnes HG, et al. Allele-specific copy number analysis of tumors. Proceedings of the National Academy of Sciences of the United States of America. 2010;107(39):16910-5.

35. Newman AM, Steen CB, Liu CL, Gentles AJ, et al. Determining cell type abundance and expression from bulk tissues with digital cytometry. Nat Biotechnol. 2019;37(7):773-82.

36. Sturm G, Finotello F, List M. Immunedeconv: An R Package for Unified Access to Computational Methods for Estimating Immune Cell Fractions from Bulk RNA-Sequencing Data. Methods Mol Biol. 2020;2120:223-32.

37. Sheffield NC, Pierron G, Klughammer J, Datlinger P, et al. DNA methylation heterogeneity defines a disease spectrum in Ewing sarcoma. Nat Med. 2017;23(3):386-95.

38. Horvath S. DNA methylation age of human tissues and cell types. Genome biology. 2013;14(10):R115.

39. Restrepo P, Bubie A, Craig AJ, Labgaa I, et al. Intra-tumoral epigenetic heterogeneity and aberrant molecular clocks in hepatocellular carcinoma. Preprint on medrxiv. 2021 [Available from: <https://www.medrxiv.org/content/10.1101/2021.03.22.21253654v1>.

40. Hua X, Zhao W, Pesatori AC, Consonni D, et al. Genetic and epigenetic intratumor heterogeneity impacts prognosis of lung adenocarcinoma. Nat Commun. 2020;11(1):2459.

41. Paradis E, Schliep K. ape 5.0: an environment for modern phylogenetics and evolutionary analyses in R. Bioinformatics. 2019;35(3):526-8.

42. Desper R, Gascuel O. Fast and accurate phylogeny reconstruction algorithms based on the minimum-evolution principle. J Comput Biol. 2002;9(5):687-705.

43. Mazor T, Pankov A, Johnson BE, Hong C, et al. DNA Methylation and Somatic Mutations Converge on the Cell Cycle and Define Similar Evolutionary Histories in Brain Tumors. Cancer Cell. 2015;28(3):307-17.

44. Liu SJ, Magill ST, Vasudevan HN, Hilz S, et al. Multiplatform Molecular Profiling Reveals Epigenomic Intratumor Heterogeneity in Ependymoma. Cell Rep. 2020;30(5):1300-9 e5.

45. Schwarz RF, Trinh A, Sipos B, Brenton JD, et al. Phylogenetic quantification of intra-tumour heterogeneity. PLoS Comput Biol. 2014;10(4):e1003535.

46. Petkovic M, Watkins TBK, Colliver EC, Laskina S, et al. Whole-genome doubling-aware copy number phylogenies for cancer evolution with MEDICC2 2021 [Available from: <https://www.biorxiv.org/content/10.1101/2021.02.28.433227v1>.

47. Watkins TBK, Lim EL, Petkovic M, Elizalde S, et al. Pervasive chromosomal instability and karyotype order in tumour evolution. Nature. 2020;587(7832):126-32.

48. Saitou N, Nei M. The neighbor-joining method: a new method for reconstructing phylogenetic trees. Mol Biol Evol. 1987;4(4):406-25.

49. Smith MR. Information theoretic generalized Robinson-Foulds metrics for comparing phylogenetic trees. Bioinformatics. 2020;36(20):5007-13.

50. Li S, Garrett-Bakelman F, Perl AE, Luger SM, et al. Dynamic evolution of clonal epialleles revealed by methclone. Genome biology. 2014;15(9):472.

51. Landan G, Cohen NM, Mukamel Z, Bar A, et al. Epigenetic polymorphism and the stochastic formation of differentially methylated regions in normal and cancerous tissues. Nature genetics. 2012;44(11):1207-14.

52. Chen X, Ashoor H, Musich R, Wang J, et al. epihet for intra-tumoral epigenetic heterogeneity analysis and visualization. Sci Rep. 2021;11(1):376.

53. Landau DA, Clement K, Ziller MJ, Boyle P, et al. Locally disordered methylation forms the basis of intratumor methylome variation in chronic lymphocytic leukemia. Cancer Cell. 2014;26(6):813-25.
