# Supplementary material for: Evidence of DNA methylation heterogeneity and epipolymorphism in kidney cancer tissue samples
Source: Oncogene. 2025 Jan 17;44(15):1024–36. doi: 10.1038/s41388-024-03270-3 (PMC11976292; doi:10.1038/s41388-024-03270-3)
Supplement: Supplementary file 2 — supplementary figures and tables [file 41388_2024_3270_MOESM2_ESM.pdf]

Supplemental Table S1: Demographic and sample data for each ccRCC patient with multi-region samples

Data are shown for 18 ccRCC patients. The number of normal (N) and tumour (T) multi-region samples are shown for each patient for methylation (Epic-seq), RNA-seq and WES. Missing samples are left blank. Clinical data are shown, including recurrence status (i.e. recurrence, no recurrence and metastases at diagnosis). One patient was lost to follow up (patient 7067). All patients had ≥4 years of follow-up.

	Characteristics at diagnosis									Follow up	Number of available samples					
Patient ID	Age (years)	T Stage	M stage	Size (cm)	Grade	Leibovich score	Necrosis	Sarcomatoid	Rhabdoid	Recurrence	Epic-seq		RNA-seq		WES	
											N	T	N	T	N	T
5532	61	pT3a	M1	6	2	4	No	No	No	Mets at Dx	1	3			1	3
5644	61	pT2a	M0	7.5	3	5	Yes	No	No	No	1	7			1	4
5790	51	pT3a	M0	6.7	3	6	Yes	No	No	No	2	3			1	2
5799	53	pT1b	M0	6.5	2	2	No	No	No	No	3	7	2	1	1	4
5801	73	pT3a	M0	10.8	4	9	Yes	Yes	Yes	Recurrence	2	4				
5802	74	pT3a	M0	2.8	3	6	Yes	No	No	No	1	3			1	2
5813	42	pT3a	M0	8.7	2	4	No	No	No	Recurrence	3	7			1	7
5818	63	pT3a	M1	7.4	3	6	Yes	No	No	Mets at Dx	1	3			1	1
5826	76	pT1b	M0	6.1	3	4	Yes	No	No	No	1	3			1	2
5842	67	pT3a	M0	13.5	4	9	Yes	No	No	Recurrence	2	9			1	8
5848	65	pT1b	M0	5.4	3	3	No	No	No	Recurrence	3	3			1	3
5998	77	pT1b	M0	5.2	2	2	No	No	No	No	3	6	2	3	1	3
6262	65	pT3a	M0	10.2	4	8	No	Yes	No	Recurrence	4	6	4	3	1	5
6285	67	pT3a	M0	7.5	4	8	Yes	No	Yes	Recurrence	2	5			1	5
6300	62	pT3b	M0	13	4	9	Yes	No	No	Recurrence	4	11	3	9	1	8
7067	56	pT3a	M0	8	3	5	No	No	No	Unknown	3	5	3	6	1	5
7068	62	pT3a	M0	12	4	9	Yes	No	No	Recurrence	2	5	2	4	1	3
7281	65	pT3b	M0	8	4	8	Yes	No	No	Recurrence	2	6	2	3	1	3

Abbreviations: M = metastases, Mets at Dx= metastases at diagnosis, N= normal, RNA-seq= RNA sequencing, T= tumour, WES = whole exome sequencing

Supplemental Table S2: Demographic and sample data for each ccRCC patient in the independent cohort (no multi-region samples)

Data are shown for 41 ccRCC patients. The number of normal (N) and tumour (T) samples are shown for each patient for methylation (Epic-seq). Clinical data are shown, including recurrence status (i.e. recurrence, no recurrence and metastases at diagnosis).

	Characteristics at diagnosis								Follow up	Number of samples	
Patient ID	T Stage	M stage	Size (cm)	Grade	Leibovich score	Necrosis	Sarcomatoid	Rhabdoid	Recurrence	Epic-seq	
										N	T
5007	pT3a	M0	4.5	2	4	No	No	No	Yes	1	1
5016	pT3a	M0	8.5	4	8	Yes	Yes	Yes	Yes	1	1
5025	pT3a	M0	10	3	6	No	No	No	Yes	1	1
5042	pT3aN1	M0	14.5	3	8	No	No	No	Yes	0	1
5396	pT3a	M0	7	3	6	Yes	No	No	Yes	1	1
5615	pT4N1	M0	17	4	9	Yes	Yes	Yes	Yes	1	1
5653	pT1b	M0	5.9	3	4	No	No	No	Yes	1	1
5835	pT3a	M0	10	2	6	Yes	No	No	Yes	0	1
6030	pT3a	M0	18	3	7	Yes	No	No	Yes	0	1
6242	pT3a	M0	8.5	4	8	Yes	No	Yes	Yes	1	1
7062	pT3a	M0	11	4	9	Yes	No	No	No	1	1
0460	pT1b	M0	6.5	3	3	No	No	No	No	0	1
0466	pT3c	M0	11	2	5	No	No	No	Yes	1	1
0467	pT3a	M0	8.2	4	8	Yes	No	No	No	0	1
0474	pT3a	M0	12	3	5	No	No	No	Yes	0	1
0481	pT3a	M0	10	3	6	Yes	No	No	Yes	0	1
0482	pT3a	M0	2.7	2	4	No	No	No	No	0	1
0486	pT3a	M0	4.5	3	5	No	No	No	No	0	1
0489	pT3a	M0	8	3	5	No	No	No	Yes	1	1
5531	pT3a	0	7	4	8	Yes	No	Yes	0	1	1
6018	pT3a	0	8	3	5	No	No	No	0	1	1
6029	pT3a	0	11.5	3	7	Yes	No	No	1	0	1
0205	pT3b	0	11.5	3	6	No	No	No	0	1	1
0227	pT3a	0	6	3	5	No	No	No	0	1	1
0261	pT3a	0	11.5	4	7	Yes	No	No	0	1	1
0290	pT3a	0	3.5	2	4	No	No	No	0	1	1
0291	pT3a	0	3.9	3	5	No	No	No	0	1	1
0306	pT3a	1	6.5	4	8	Yes	Yes	No	Mets at Dx	0	1
0309	pT3a	0	7	2	4	No	No	No	0	1	1
0322	pT3b	0	10	3	7	Yes	No	No	1	0	1
5022	pT3a	0	6	3	5	No	No	No	0	1	1
5021	pT3a	0	4.5	2	4	No	No	No	0	1	1

5533	pT3a	0	13	4	8	Yes	No	Yes	0	1	1
5807	pT3a	0	7.5	3	5	No	No	No	0	1	1
5991	pT3a	0	7	2	4	No	No	No	0	1	1
6023	pT3a	0	8.5	3	6	Yes	No	No	0	1	1
6025	pT3a	0	2.5	2	4	No	No	No	0	1	1
5604	pT3a	0	8.5	2	4	No	No	No	0	1	1
5006	pT3a	0	4.7	3	5	No	No	No	0	1	1
5634	pT3aN1	1	11.5	4	11	Yes	No	No	Mets at Dx	1	1
5626	pT3a	1	4.5	2	4	No	No	No	Mets at Dx	1	1

Abbreviations: M = metastases, Mets at Dx= metastases at diagnosis, N= normal, T= tumour.

Supplemental Table S3: Robinson-Fould distance

Robinson-Fould distance comparing phylogenies derived using DNA methylation and copy number data.

Patient ID	Robinson-Fould distance
5644	0.77
5813	0.86
5842	0.69
6262	0.75
6285	0.87
6300	0.59
7067	0.49
5532	0 (identical trees)

Supplemental Table S4: Genes known to be associated with ccRCC which demonstrate differential epipolymorphism

Genes known to be associated with ccRCC that were found to have significant differential epipolymorphism within their promoter region in ccRCC versus normal kidney tissue.

	Identified in the original cohort of ccRCC vs normal tissue (135 multiregion samples)	Genes with differential epipolymorphism in external validation cohort of ccRCC vs normal tissue (71 samples)
Genes with significant differential epipolymorphism in their promoter region	47 genes: *MAD2L2, AGT, CD44, KRT18, TRPC4, NDRG2, CD276, NOS2, TTYH2, RAB37, FRZB, TERT, TBXT, MUC3A, PRKCZ, TP53BP2, TGFBR3, HIF1AN, FGFR2, ALOX5, ADM, IL18, ST3GAL4, WT1, PXN, KRT7, WIF1, MOK, PRKCH, IGF1R, ABCC1, CXCL16, HPN, CGB3, BSG, BCL2L11, JAG1, EGF, DUSP1, SOD2, HLA-G, HLA-A, TAP2, PODXL, MAD1L1, IGF2BP3, CCN3, MYC*	31 genes: *MAD2L2, WT1, CD44, TRPC4, NDRG2, CD276, NOS2, TTYH2, RAB37, TERT, PRKCZ, AGT, FGFR2, ADM, IL18, WT1, KRT7, WIF1, PRKCH, IGF1R, ABCC1, BSG, BCL2L11, JAG1, EGF, SOD2, HLA-G, HLA-A, TAP2, MAD1L1, MYC*

Supplemental Table S5: Linear models to predict gene expression, for e-loci with significantly higher epipolymorphism in ccRCC

Linear models to predict gene expression based on methylation and epipolymorphism versus methylation alone, for e-loci with significantly higher epipolymorphism in ccRCC compared to normal tissue. The likelihood ratio test was used to compare adjusted R^2 values for the two models. Where multiple e-loci were found to be significant, the number of significant e-loci are reported and the e-locus with the lowest adjusted p value is shown in the table. Genes are ranked based on descending adjusted R^2 for the linear model using methylation and epipolymorphism.

Gene	Correlation coefficient: Epipolymorphism vs gene expression	Linear model adjusted R^2, Gene expression predicted by methylation	Linear model adjusted R^2, Gene expression predicted by methylation and epipolymorphism	Likelihood ratio test adjusted p value	Number of significant e-loci in the promoter region of the gene
RASL11B	-0.78	0.42	0.62	0.005	2
ZNF728	-0.78	0.45	0.59	0.029	2
DPP6	-0.66	0.14	0.58	0.0003	4
KRBA1	0.76	0.31	0.56	0.004	4
HS3ST3B1	-0.73	0.3	0.53	0.006	5
KRT18	0.73	0.37	0.51	0.044	1
ICA1	-0.68	0.21	0.47	0.007	1
LINC02693	-0.66	0.29	0.46	0.03	1
DLGAP1	-0.66	0.17	0.44	0.007	3
LRAT	0.60	0.16	0.44	0.007	4
ESPNP	-0.59	0.16	0.42	0.009	2
MAN1C1	-0.67	0.26	0.42	0.049	1
CD4	0.66	-0.02	0.41	0.004	1
MIR663AHG	-0.46	-0.02	0.40	0.007	1
PLAC9	-0.40	-0.01	0.33	0.015	1
LINC01287	0.14	0.07	0.30	0.044	1
PRR5L	-0.39	-0.01	0.28	0.009	3
ANKRD18CP	-0.31	-0.02	0.27	0.012	1
SLC22A31	-0.37	-0.02	0.27	0.01	2
CD8B2	-0.24	-0.01	0.24	0.03	1
PRDM6	-0.40	-0.02	0.23	0.031	2

Supplemental Table S6: Linear models to predict gene expression, for e-loci with significantly higher epipolymorphism in normal kidney

Linear models to predict gene expression based on methylation and epipolymorphism versus methylation alone, for e-loci with significantly higher epipolymorphism in normal kidney compared to ccRCC. The likelihood ratio test was used to compare adjusted R^2 values for the two models. Where multiple e-loci were found to be significant, the number of significant e-loci are reported and the e-locus with the lowest adjusted p value is shown in the table. Genes are ranked based on descending adjusted R^2 for the linear model using methylation and epipolymorphism. A total of 82 genes were identified (adjusted p value <0.05), however only the top-most genes are shown here.

Gene	Correlation coefficient: Epipolymorphism vs gene expression	Linear model adjusted R^2, Gene expression predicted by methylation	Linear model adjusted R^2, Gene expression predicted by methylation and epipolymorphism	Likelihood ratio test adjusted p value	Number of significant e-loci in the promoter region of the gene
SLC16A3	-0.92	0.77	0.84	0.001	3
H2BC13	-0.69	0.21	0.63	7.67E-06	4
MFHAS1	0.70	0.24	0.63	8.20E-06	8
H2AC11	-0.72	0.11	0.54	1.14E-05	5
PDZD2	0.54	0.19	0.51	0.001	1
B3GNTL1	-0.69	0.25	0.47	0.005	1
CLDN4	0.39	0.25	0.46	0.005	3
H2AC16	-0.20	-0.01	0.45	2.69E-05	8
H2BC11	-0.67	0.26	0.44	0.009	2
LPCAT1	-0.60	0.01	0.44	6.08E-05	2
UBE2D2	-0.67	0.08	0.44	1.86E-04	10
NINJ2	-0.68	0.06	0.43	0.001	5
IGF2BP3	-0.63	0.13	0.42	0.002	1
NFASC	0.56	0.02	0.41	0.0003	1
RRM2	-0.54	0.09	0.38	0.002	3
METRNL	0.20	0.05	0.35	0.003	3
TUBBP5	0.51	-0.01	0.35	0.001	2
SMTNL2	0.59	-0.02	0.33	0.002	12
SNX29P2	0.54	0	0.33	0.004	1
ATAD5	-0.51	0.1	0.32	0.009	2
MSC	-0.55	0.02	0.32	0.009	2
OLFML2A	0.24	0.06	0.32	0.005	2
UBC	-0.54	0.04	0.32	0.004	4
TBC1D14	0.56	-0.01	0.31	0.002	2
ZNF888	-0.56	0.02	0.3	0.004	1
LMF1-AS1	0.53	0.01	0.26	0.009	1

Supplemental table S7: Summary of results of latent methylation components analysis

Latent methylation components (LMCs) are shown. All correlations (cor) shown in the table had an adjusted p value < 0.001.

	Hypothesis	Correlation with purity estimates (RNA-seq, Epic-seq and WES)	Correlation with reference methylomes
LMC1	Represents immune cell, likely T cells	LMC1 was correlated with the immune score from RNA-seq (cor=0.60)	LMC1 was correlated with T cells (cor=0.83) and other immune cells to a lesser degree
LMC2	Cell type unclear		
LMC3	Represents immune cell, likely tumour associated macrophages	LMC3 was correlated with the immune score from RNA-seq (cor=0.50)	LMC3 was correlated with monocytes (cor = 0.96) and neutrophils (cor=0.95)
LMC4	Represents kidney epithelium in ccRCC tumours	LMC4 was correlated with RNA-seq purity estimates for tumour samples (cor = 0.62) and there was no correlation with purity estimates for normal kidney samples. LMC4 was also correlated with purity estimates from WES (cor=0.49).	
LMC5	Represents normal (non-cancerous) kidney epithelium	LMC5 was correlated with RNA-seq purity estimate in normal tissue (cor=0.72) and there was no correlation with purity estimates for ccRCC tumour samples. LMC5 was also negatively correlated with Infiniumpurify (cor=-0.71) which measures contamination with normal kidney.	
LMC6	Cell type unclear		
LMC7	Cell type unclear		

Supplemental Figure S1: Methylation heterogeneity between patients

Principal component analysis (PCA) demonstrating normal kidney and ccRCC samples (figure A). PCA demonstrating normal kidney and ccRCC samples, highlighting multi-region tumour samples from the same patient (figure B). Heatmaps visualising methylation values in the top 10,000 CpGs (figure C) and top 50,000 CpGs (figure Panel D) with the highest variance in tumour samples. The top annotation demonstrates which multi-region tumour samples are derived from the same patient, along with tumour purity (estimated using WES or RNA-seq).

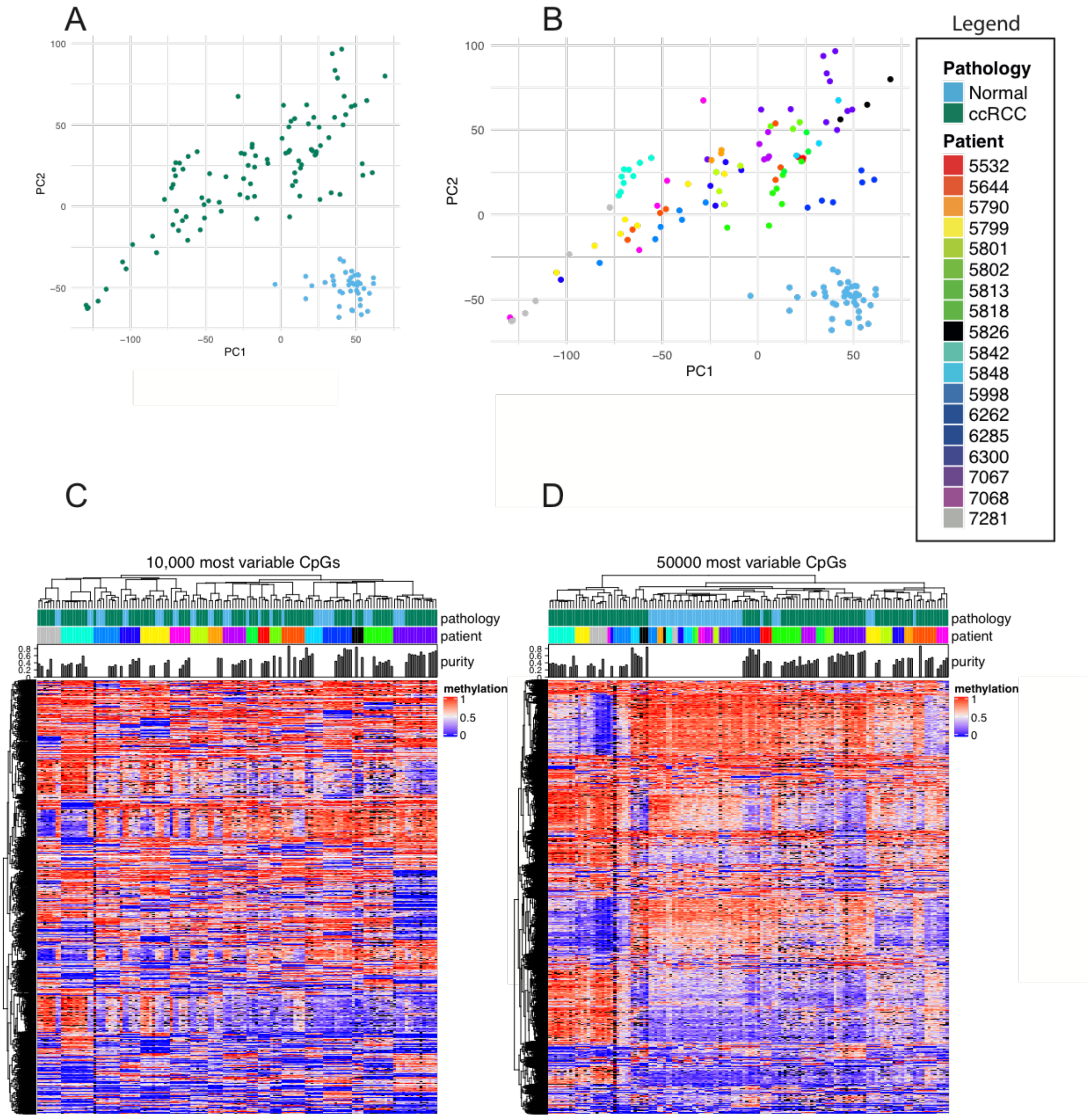

Supplemental Figure S2: DNA methylation age versus chronological age in ccRCC and normal kidney samples in TCGA and our dataset

There is an obvious correlation between DNA methylation age and chronological age, which is more pronounced in normal tissue than ccRCC (figure A). DNA methylation age is significantly higher in ccRCC compared to normal kidney samples in TCGA and my dataset (p value <0.01; figure B). The predicted to chronological age ratio (PCAR) is shown for each sample, by patient (figure C). Tumour samples tend to have a higher PCAR than normal kidney. The dotted horizonal line represents a PCAR of 1, i.e. the DNA methylation and chronological age are the same.

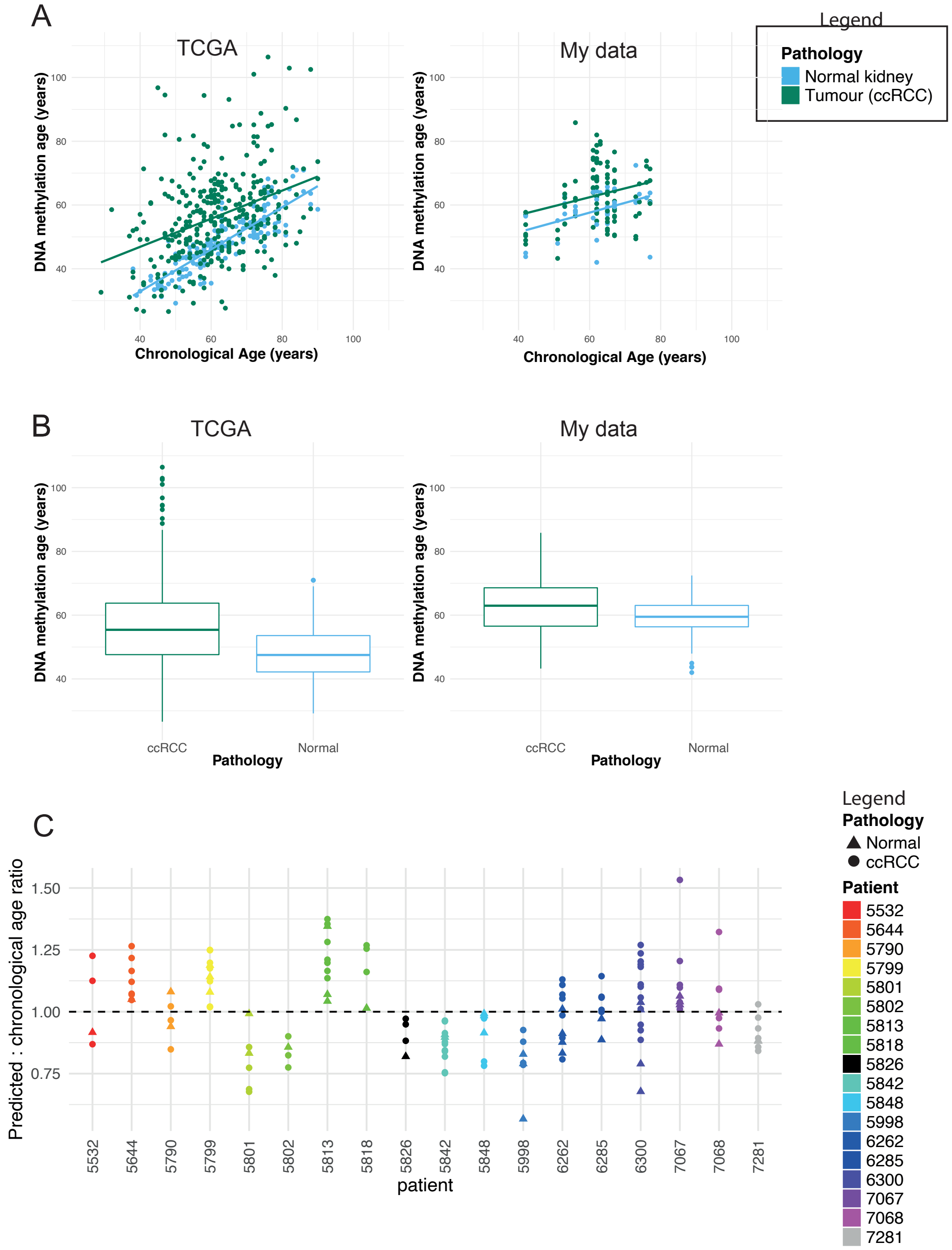

Supplemental Figure S3: mutation, copy number and methylation analysis

Oncoplot demonstrating mutational status for each sample, for top driver genes in renal cancer [1] (figure A). Copy number status is also shown for chromosome 3p, the most commonly affected copy number aberration. Heatmap of DNA methylation changes and mutational status of the *VHL* gene (figure B). Each column represents a patient sample, and each row represent a CpG within the *VHL* gene (all annotated CpGs are shown ordered by genomic position starting at the transcription start site). The top annotation track demonstrates the sample pathology (tumour vs normal), patient ID, recurrence status and *VHL* gene mutational status. The dendrogram demonstrates two clear branches, with samples from patient 5826 represented on the left side of the branch, separate from other samples.

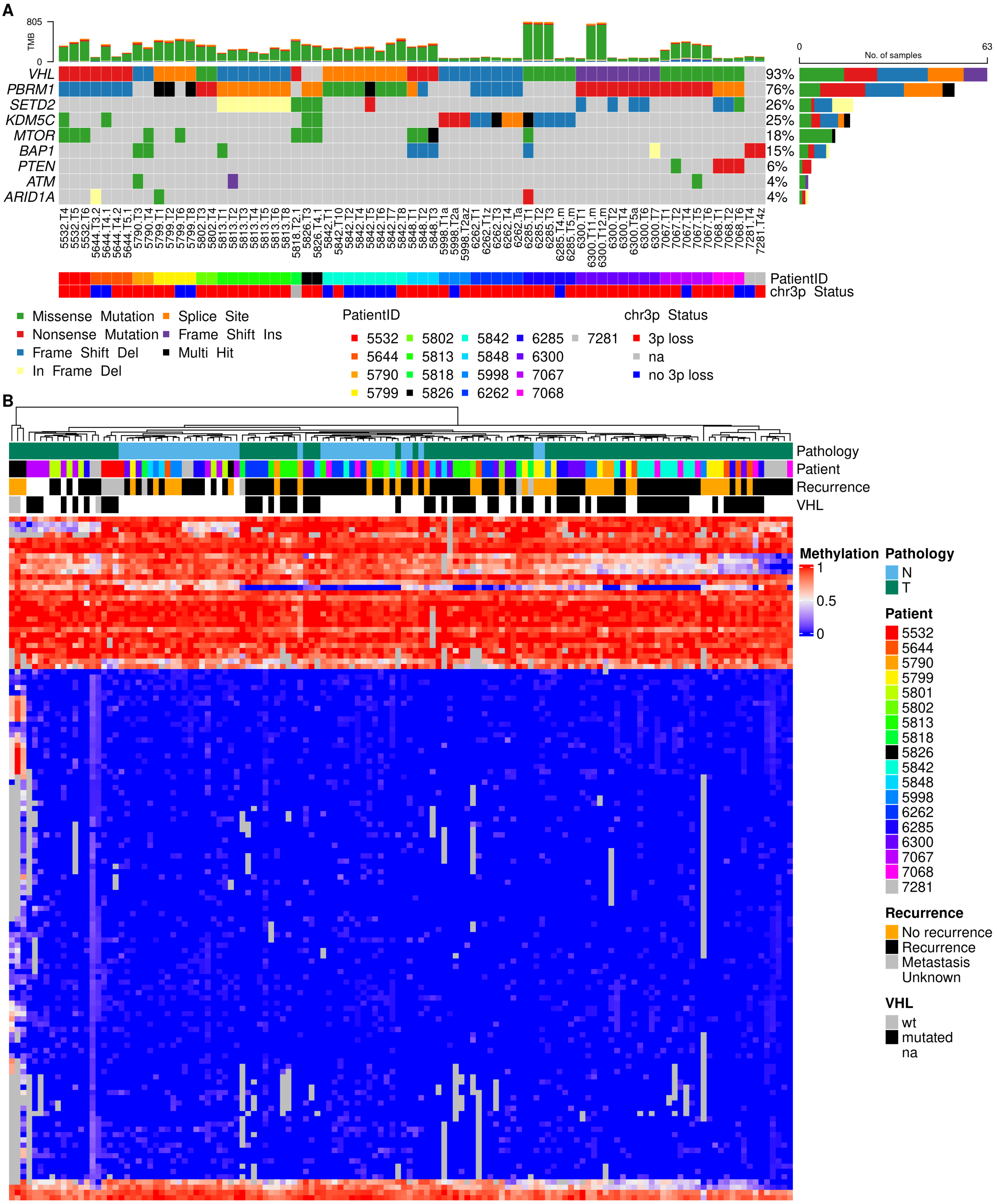

Supplemental Figure S4: Average Pairwise ITH (APITH) index

Scatterplot of APITH derived using methylation versus copy number data. There was no significant correlation.

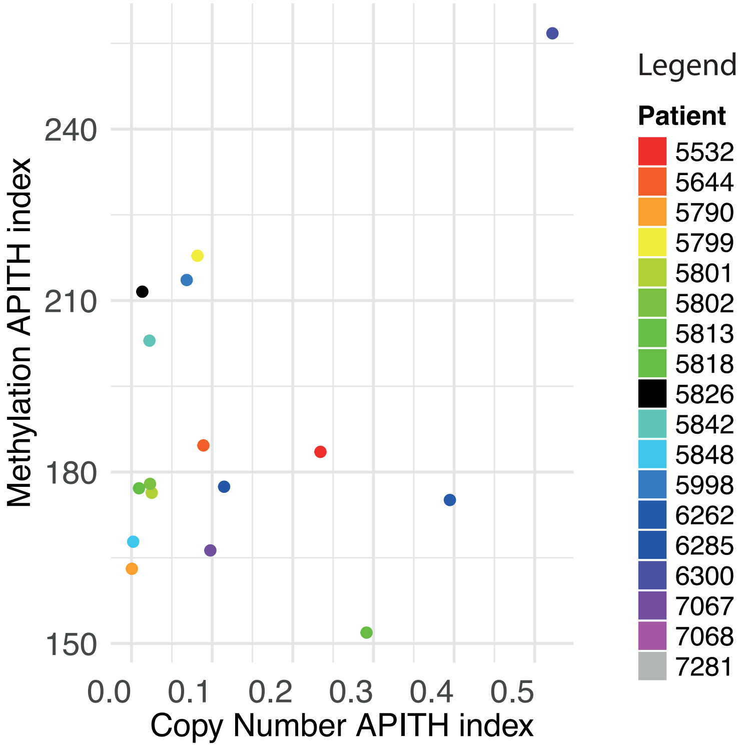

Supplemental Figure S5: Phylogenies for patients 5644, 5813, 5842 and 5532

Phylogenies using DNA methylation and copy number data are compared for each patient, for patients 5644, 5813, 5842 and 5532. Monophyletic clades which are present in both phylo-epigenetic and phylogenetic trees are shown in red, with other similarities shown in green.

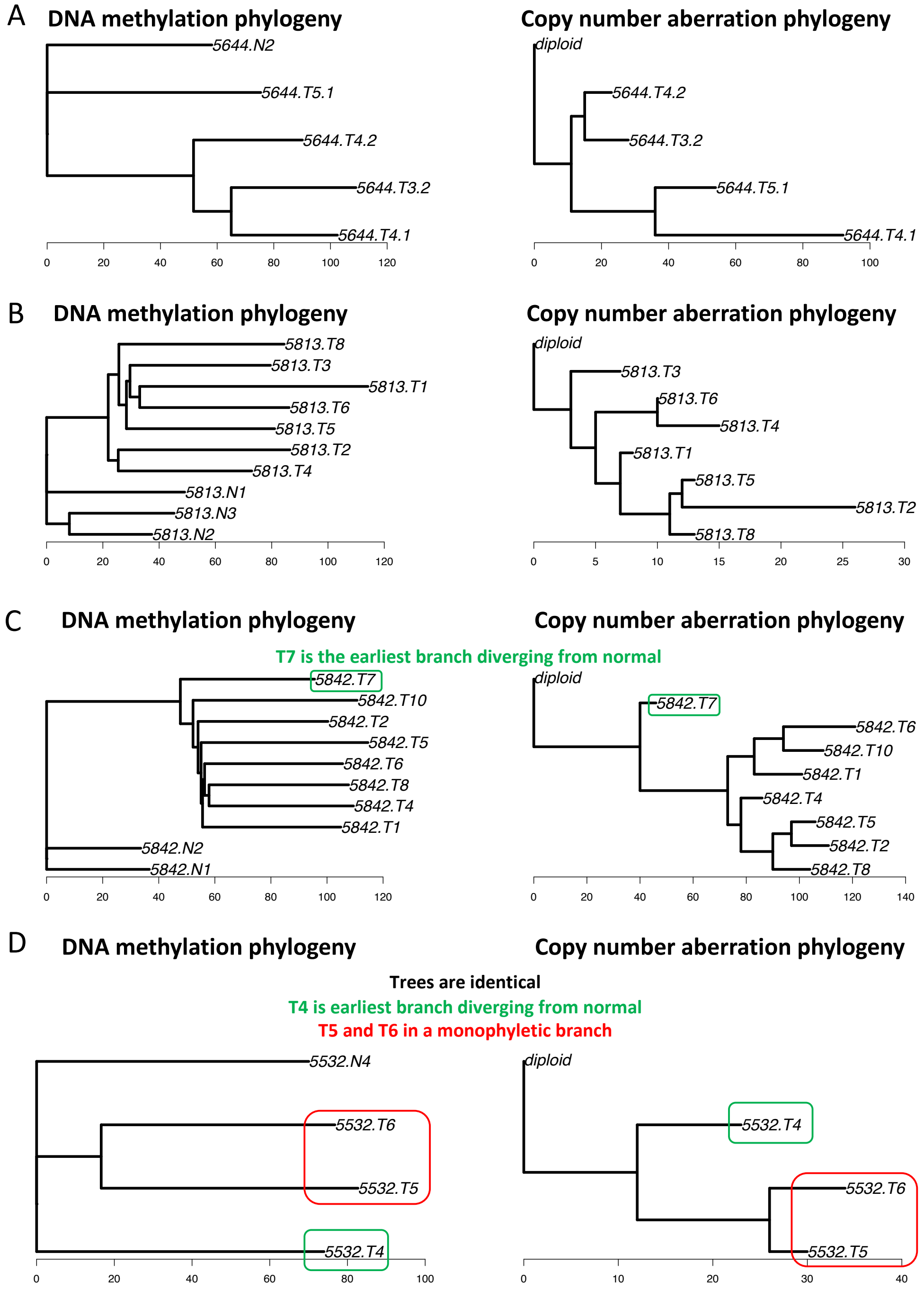

Supplemental Figure S6: Phylogenies for patients 6285, 6300, 7067 and 6262

Phylogenies using DNA methylation and copy number data are compared for each patient, for patients 6285, 6300, 7067 and 6262. Monophyletic clades which are present in both phylo-epigenetic and phylogenetic trees are shown in red, with other similarities shown in green.

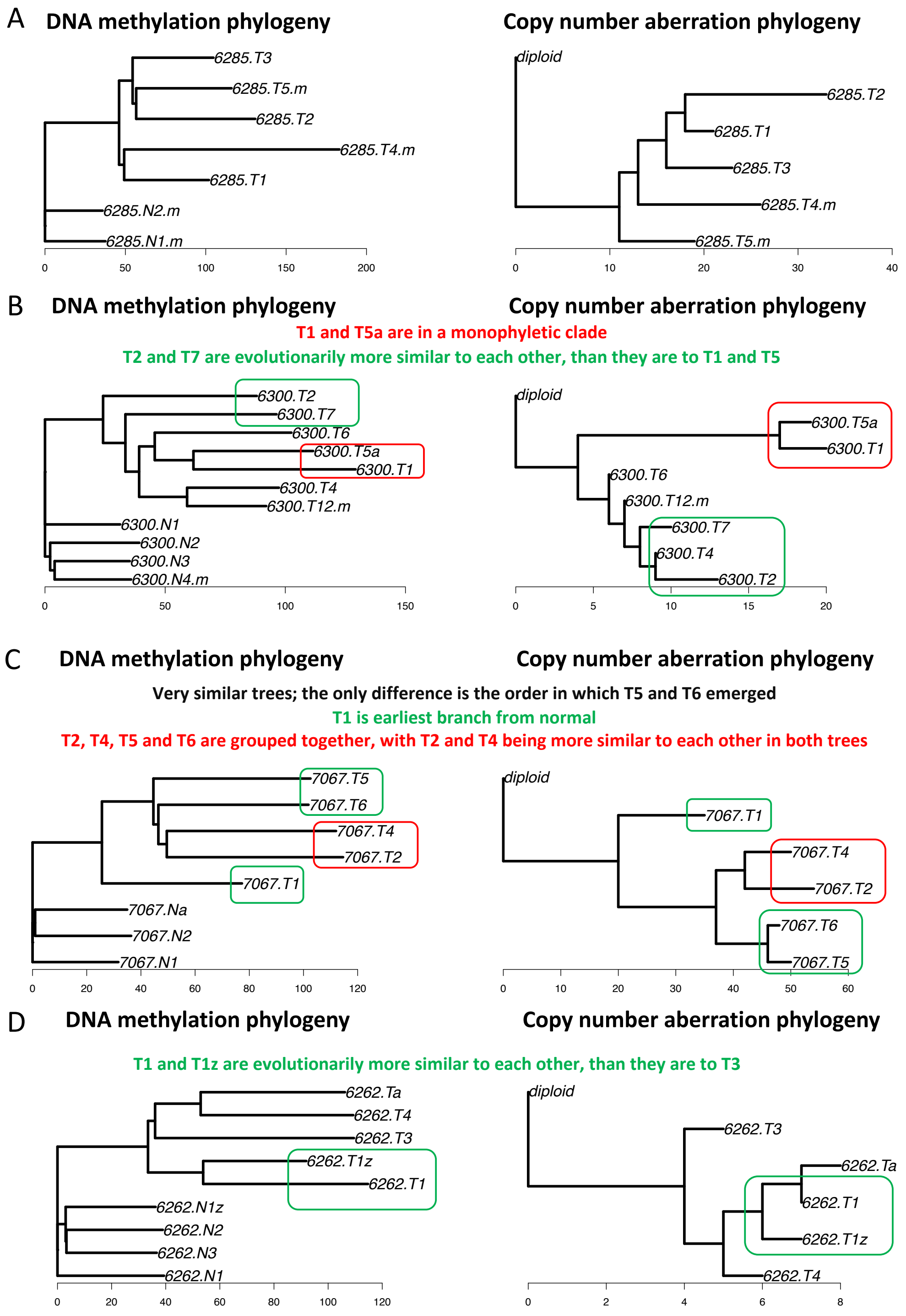

Supplemental Figure S7: CpG island methylator phenotype (CIMP)

Heatmap visualising methylation values for tumour and normal samples for CIMP CpGs derived using methods described by Arai et al [2] (Figure A) and methods used in TCGA [3] (Figure B). Each column represents a patient sample, and each row represent a CpG (or probes on the Illumina array). The top annotation track demonstrates the sample pathology (tumour vs normal), patient ID and recurrence status.

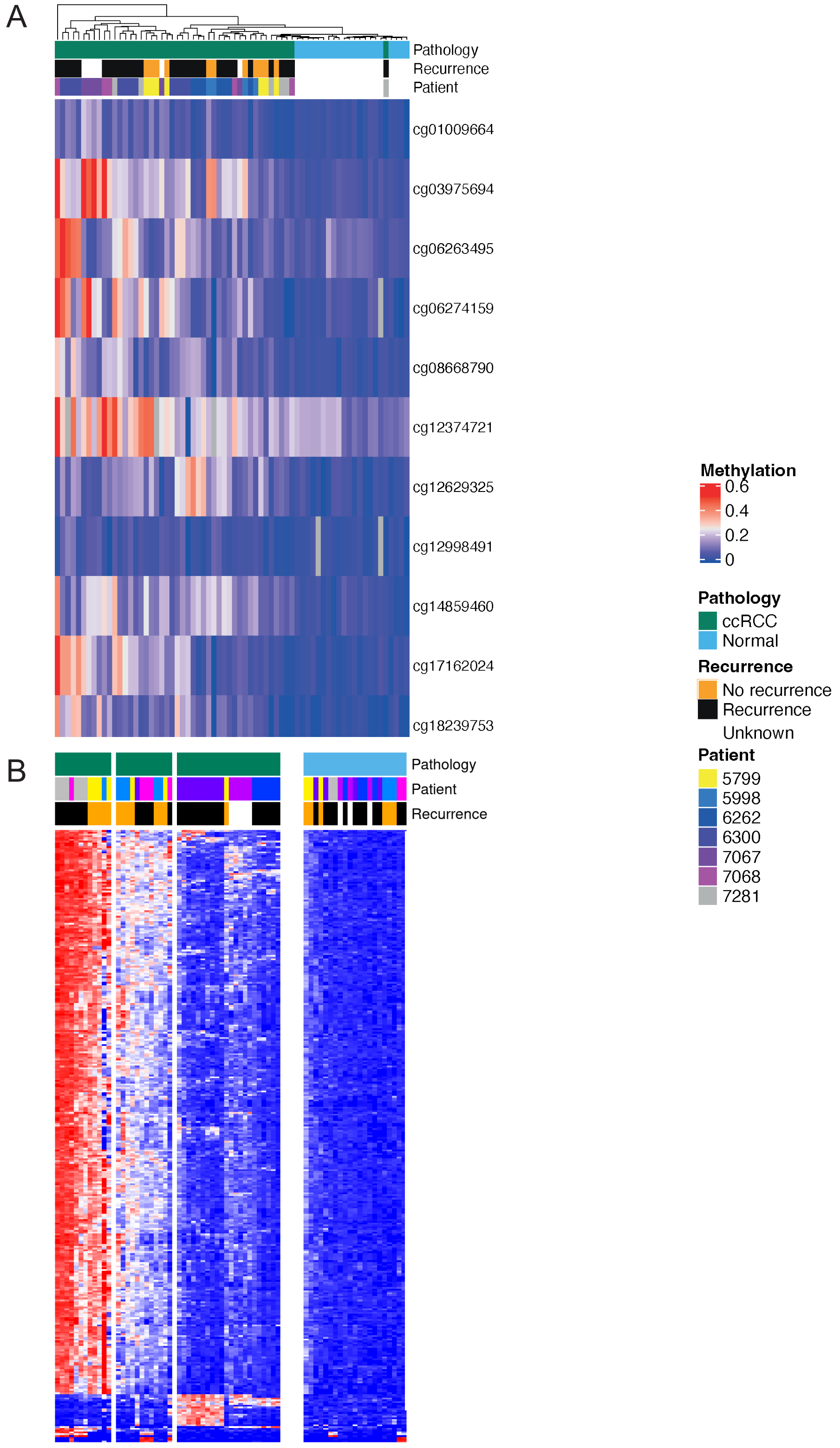

Supplemental Figure S8: Epipolymorphism versus average methylation

Scatterplot of epipolymorphism versus average methylation at e-loci for the entire dataset (N= 138,412 e-loci). The red lines represent density plot contours, demonstrating that the majority of points display low epipolymorphism and methylation levels of 0 or 1. The grey line highlights the U-shaped distribution.

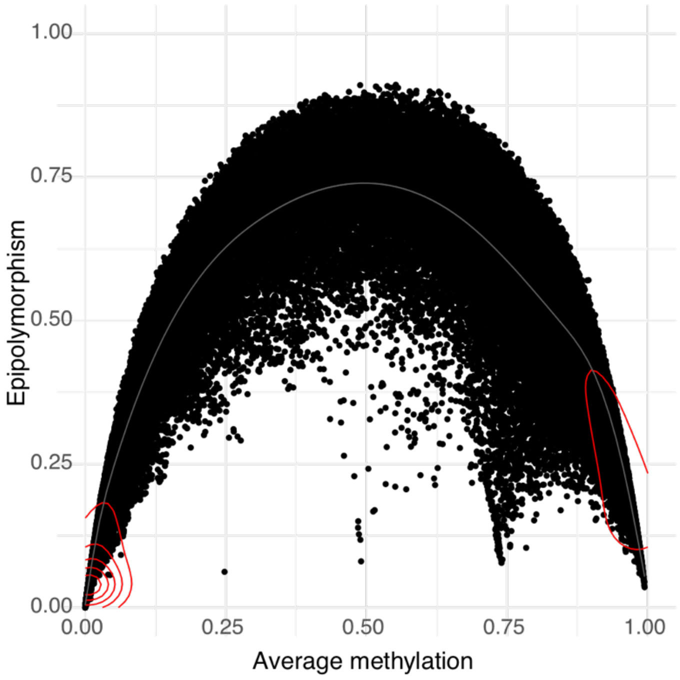

Supplemental Figure S9: Epipolymorphism in normal kidney, ccRCC tissue and OS-RC-2 ccRCC cell line

Epipolymorphism is shown in ccRCC tumour tissue, normal kidney tissue and the OS-RC-2 renal cancer cell line for: all e-loci (A), e-loci with significantly higher epipolymorphism in normal kidney (B) and e-loci with significantly higher epipolymorphism in ccRCC (C). Panel D demonstrates epipolymorphism values for selected e-loci in the promoter region of 8 genes which are known to be associated with kidney cancer and were found to have significantly higher epipolymorphism in ccRCC vs normal kidney.

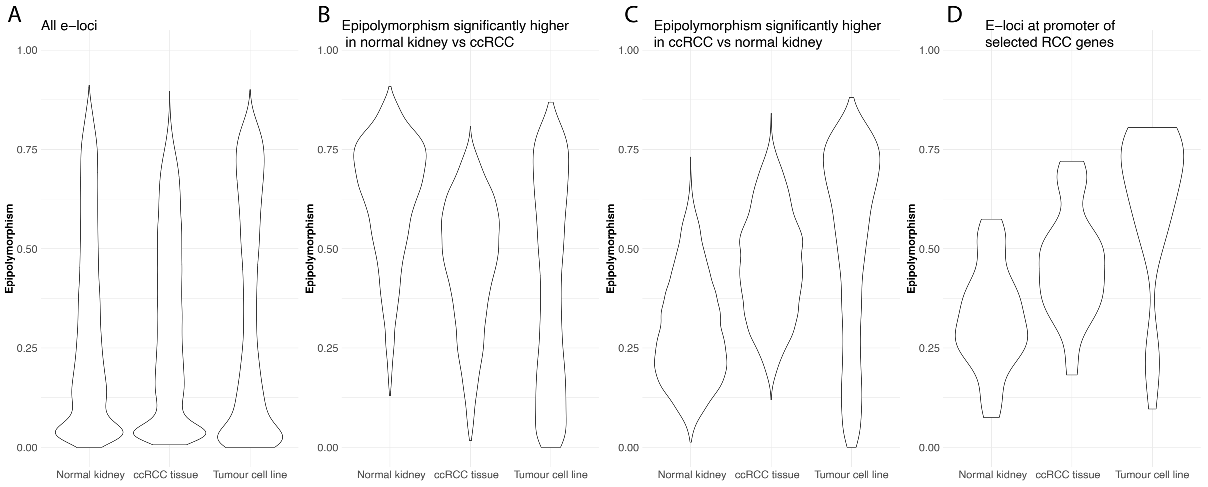

Supplemental Figure S10: DMCs with low and high variance in tumours

Heatmap of methylation levels for ccRCC and normal kidney at selected differentially methylated cytosines (DMCs). DMCs were identified and the top 10% with the lowest variance in tumour samples (A) were compared to the top 10% of DMCs with the highest variance in tumour samples (B). The top annotation bar shows tumour purity for each sample (estimated using WES or RNA-seq). Tumour purity for ccRCC samples which clustered either with normal samples, or away from normal samples in the dendrogram accompanying the heatmap in Panel B (C). Tumour purity for each sample was calculated using WES or RNA-seq. Purity was significantly higher in samples that cluster more closely with normal tissue than those that cluster away from normal (mean purity 59.4% vs 39.9%, p value = 3.9e-8).

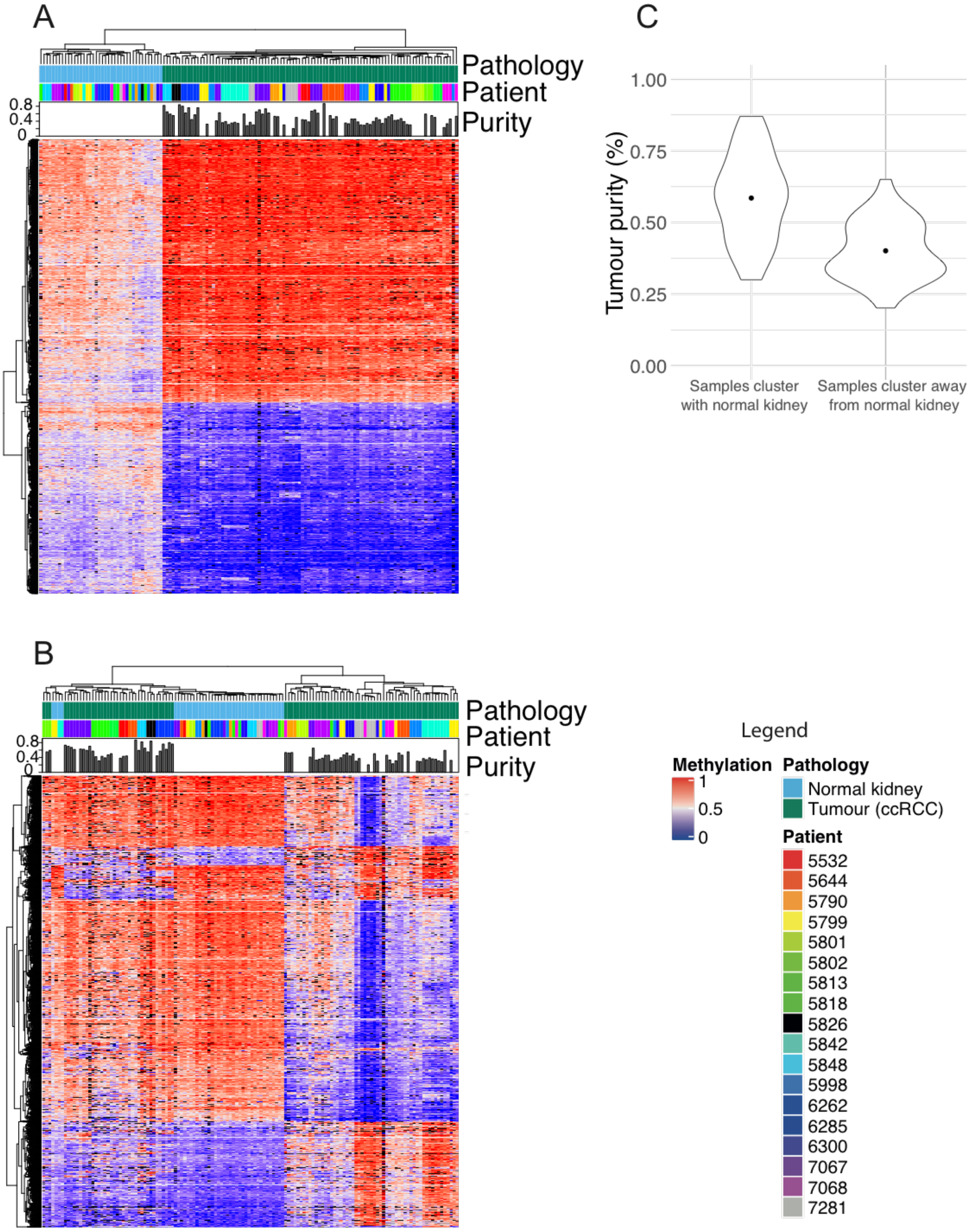

Supplemental Figure S11: Purity assessment in multi-region samples

Schematic summarising three orthogonal methods used to derive purity estimates: DNA methylation analysis using 'InfiniumPurify', RNA-seq using 'ESTIMATE' and whole exome sequencing (WES) using 'ASCAT' (A). ESTIMATE scores, immune scores and stromal scores generated using RNA-seq data and the 'ESTIMATE' package for TCGA and our data (B). Scatterplot of matched purity values for my samples obtained using RNA-seq and WES (C). Pearson correlation coefficient = 0.81

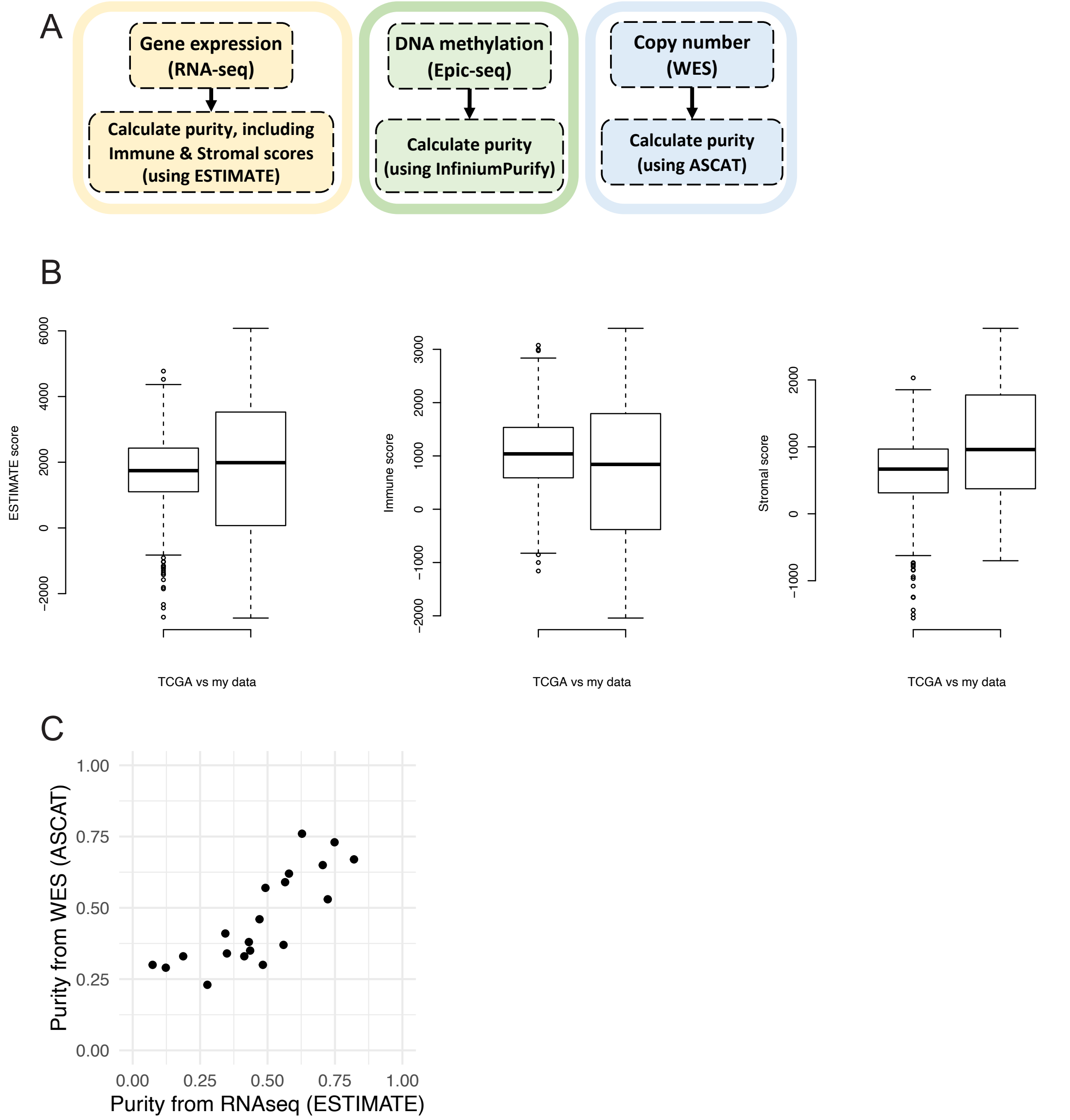

Supplemental Figure S12: LMC1 content in tumour samples with and without necrosis

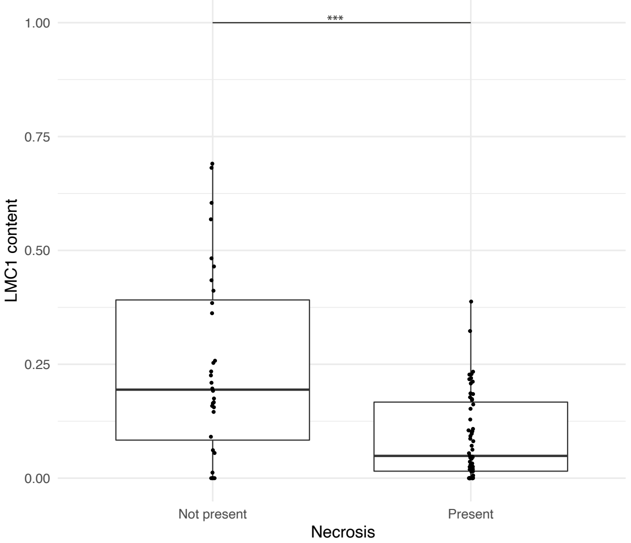

References

1. Martincorena I, Raine KM, Gerstung M, Dawson KJ, et al. Universal Patterns of Selection in Cancer and Somatic Tissues. Cell. 2018;173(7):1823.

2. Arai E, Chiku S, Mori T, Gotoh M, et al. Single-CpG-resolution methylome analysis identifies clinicopathologically aggressive CpG island methylator phenotype clear cell renal cell carcinomas. Carcinogenesis. 2012;33(8):1487-93.

3. Cancer Genome Atlas Research N, Linehan WM, Spellman PT, Ricketts CJ, et al. Comprehensive Molecular Characterization of Papillary Renal-Cell Carcinoma. The New England journal of medicine. 2016;374(2):135-45.
